# Activity assays for flavoprotein oxidases: an overview

**DOI:** 10.1007/s00253-025-13494-2

**Published:** 2025-05-08

**Authors:** Lars L. Santema, Marco W. Fraaije

**Affiliations:** Molecular Enzymology, University of Groningen, Nijenborgh 3, 9747 AG Groningen, The Netherlands

**Keywords:** Oxidase activity assays, Horseradish peroxidase, Chromogenic assays, Electrochemical assays, Photometric assays

## Abstract

**Abstract:**

Flavoprotein oxidases have found many biotechnological applications. For identifying and improving their characteristics, it is essential to have reliable and robust assay methodology available. The methodologies used to monitor their activity seem to be scattered in the literature and seem often selected based on convenience. Due to the diversity of reactions catalyzed by flavoprotein oxidases, it is virtually impossible to recommend a single activity assay. A literature analysis of 60 recent papers describing flavoprotein oxidases revealed that continuous spectrophotometric assays, in particular colorimetric assays, are the preferred choice, as they are facile, scalable and allow for better interpretation of data than discontinuous assays. Colorimetric assays typically rely on the extinction coefficient of a monitored chromogenic product, which can be highly variable depending on the experimental conditions. Therefore, it is important to determine the extinction coefficient under the specific experimental conditions used, rather than taking it directly from the literature. To provide a guideline and assist in standardization, this review describes the most commonly utilized activity assays for flavoprotein oxidases, along with their respective merits and limitations.

**Graphical Abstract:**

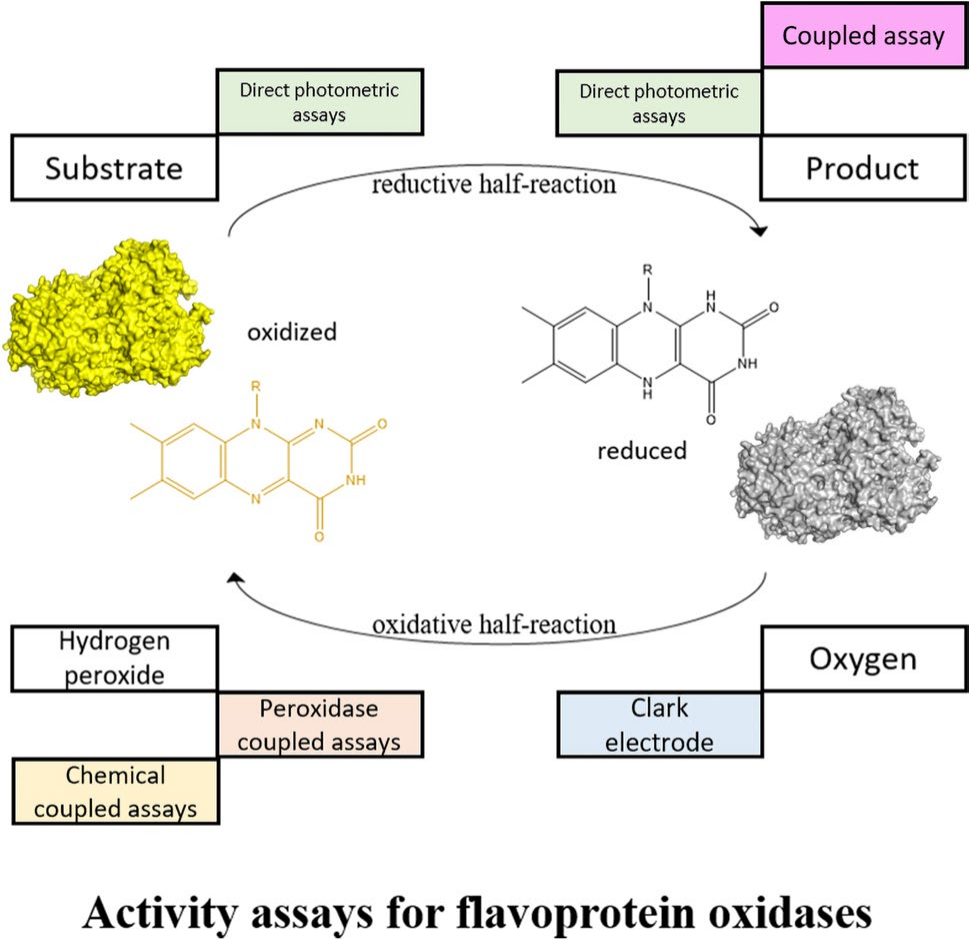

**Key points:**

• *Researchers should be more aware of limitations of activity assays.*

• *Extinction coefficients should be determined for the appropriate experimental setup.*

• *New robust activity assays are desired.*

**Supplementary Information:**

The online version contains supplementary material available at 10.1007/s00253-025-13494-2.

## Introduction

Over the past century, the utilization of enzymes as catalytic tools in biotechnology has significantly increased, substituting or enhancing traditional chemical processes across various industries (Chapman et al. 2018; Robinson 2015). Additionally, enzymes have found applications in other areas, such as in food processing and (bio)sensing (Kurbanoglu et al. 2020; Sindhu et al. 2020). The remarkable selectivity of enzymes, coupled with their capability to operate under mild conditions, has driven their widespread adoption (Chen & Arnold 2020). The interest in developing enzyme-based processes has especially increased due to recent efforts to achieve climate neutrality and replace unsustainable chemical process (Aslam et al. 2020; Chen et al. 2023; Cipolatti et al. 2019).

Redox enzymes form the class of enzymes that are capable of performing oxidations and reductions, often displaying exquisite regio- and/or enantioselectivity. A major group of redox enzymes can be classified as flavoenzymes: enzymes that contain a flavin cofactor to facilitate redox catalysis.

Flavoenzymes are one of the most utilized type of enzymes, with their application spanning various industries since the 1970 s (Pimviriyakul & Chaiyen 2020). Flavoprotein oxidases, which merely rely on molecular oxygen as electron acceptor, are perhaps the most cost-effective redox enzymes. In contrast with many other redox enzymes, these biocatalysts do not depend on (expensive) coenzymes and solely produce hydrogen peroxide as a by-product (Martin et al. 2020). This by-product can be easily eliminated by the use of a catalase thereby regenerating some molecular oxygen. In other applications, the produced hydrogen peroxide itself is the desired product or it can also be utilized for completing a cascade reaction that involves a peroxide-dependent enzyme (Habib et al. 2017). Examples of flavoprotein oxidase-based applications can be found in glucose biosensors (Mandpe et al. 2020), valorization of waste products (Binoy et al. 2022), and discovery of therapeutic inhibitors (Youdim et al. 2006). A famous example of an industrially widely applied flavoprotein oxidase is glucose oxidase. This oxidase was one of the first oxidases adopted in biotechnological processes several decades ago and can nowadays be found in numerous applications (Wong et al. 2008).

All-in-all, the above illustrates the importance of and interest in flavoprotein oxidases. For developing or improving oxidase-based applications, experimental research on flavoprotein oxidases is an active field and reliable assays to monitor oxidase activity are indispensable. Whereas the interpretation and documentation of flavoprotein oxidase activity is well addressed in literature (Bisswanger 2014; Lauterbach et al. 2023; Pleiss 2021; Schomburg et al. 2002) and an ongoing endeavor (Apweiler et al. 2010; Range et al. 2022), the various different methodologies for obtaining said data can only be found scattered throughout literature. Despite the existence of some excellent books on the topic (Bisswanger 2019; Egbuna et al. 2022; Eisenthal & Danson 2002) and reviews about specific oxidases (Reis & Binda 2023; Rosini et al. 2018), the choice for a certain method often appears to depend on the availability and convenience for the researcher. A thorough comparison of described methods seems to be missing while a well-informed choice for a certain activity assay could benefit research. With this review, we attempt to give an overview of the commonly used flavoprotein oxidase activity assays. With a critical analysis of the pros and cons of the various approaches, it may serve as a guide for choosing a particular assay for research.

**Fig. 1:**
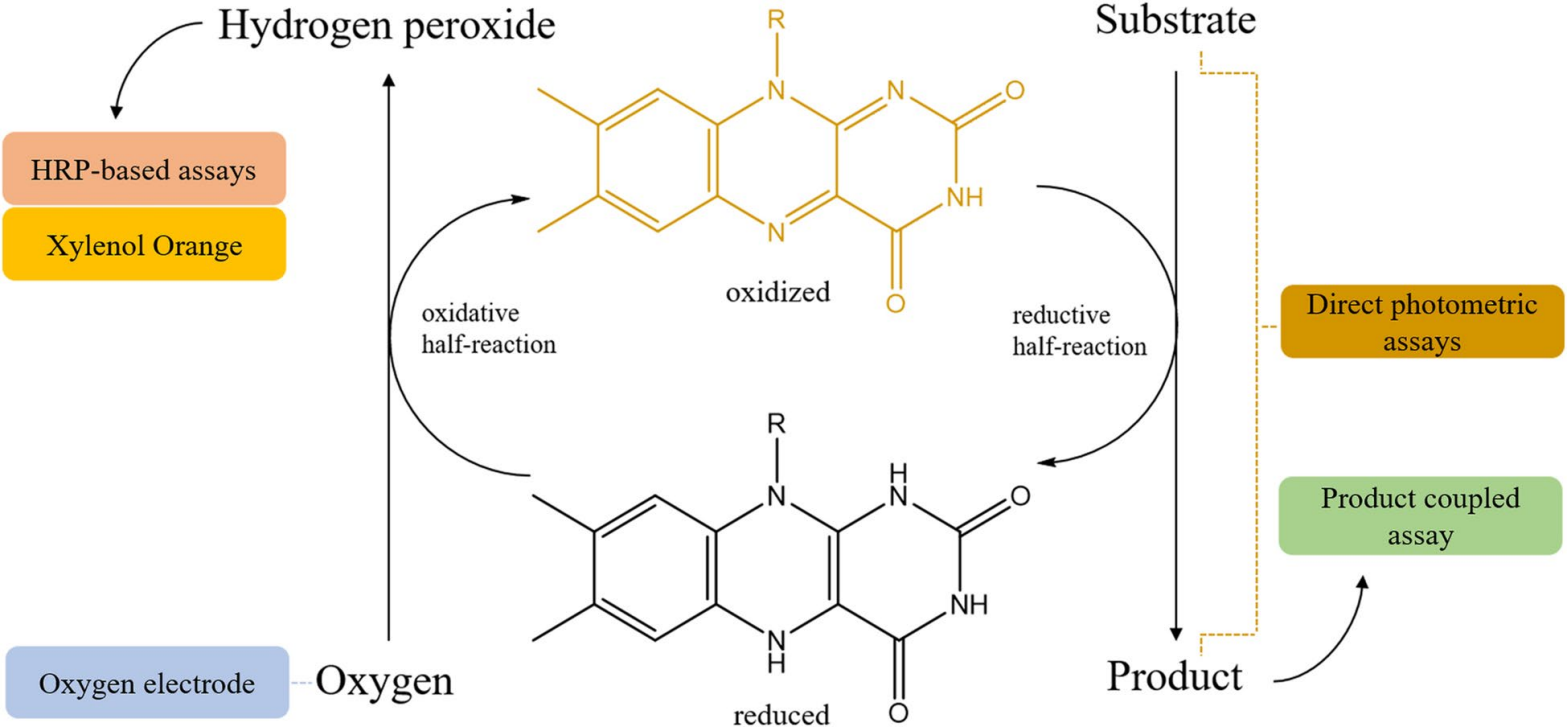
Simplified representation of a flavoprotein oxidase-catalyzed reaction and the respective activity assays described in this review. The chemistry behind each type of assay is indicated

In the last decades, a variety of oxidase assays has been reported in literature. While some of these assays may also be applicable for other types of redox enzymes, such as copper-dependent oxidases, we focus our review on assays that were demonstrated with one or more flavoprotein oxidases. An analysis of 60 recent publications (since 2020) detailing flavoprotein oxidases reveals a wide array of different activity assays employed (SI1). Most of the described assays are based on one of the following three specific catalytic properties of the oxidases: (1) the production of hydrogen peroxide (Keston 1956), (2) the consumption of molecular oxygen (Smith & Camerino 1963), or (3) product formation (De Jong et al. 1992) (Fig. 1). The analysis revealed that the majority of assays utilized are continuous (51 out of 60), allowing for real-time monitoring of the reaction, rather than discontinuous, which involve quenching the reaction at specific intervals (Harris & Keshwani 2009). This observation aligns with existing literature recommending continuous assays, as assays can exhibit lag or burst phases due to hysteretic effects (Eisenthal & Danson 2002; Frieden 1979). These artifacts can be accounted for and corrected in continuous assays, but they often remain undetected in discontinuous assays. Additionally, spectrophotometric-based assays are the most frequently used (55 of 60), most likely due to their user-friendly nature. Recommending a specific assay is nearly impossible as a choice needs to be based on sensitivity, cost, convenience, specifics of the reaction and many more aspects. Nonetheless, a good understanding of the pros and cons of the available assays is important to efficiently perform research, such as efforts in directed evolution and high-throughput screening of flavoprotein oxidases (Atkin et al. 2008; Ebrahimi Fana et al. 2023; Rembeza et al. 2022).

The accuracy of these high-throughput screening campaigns is often dependent on the precision of the activity assay used, highlighting the importance of a well-thought-out decision for the utilized activity assay (Wang et al. 2021). To provide a guideline in choosing an appropriate flavoprotein oxidase assay, this review aims to describe the most used spectrophotometric and electrochemical assays. The information on these oxidase activity assays may also be used for other oxidases, such as copper-dependent oxidases. Other types of activity assays such as calorimetry, radiometric, and liquid chromatographic assays fall out of the scope of this review and are well-documented elsewhere (Eisenthal & Danson 2002; Falconer et al. 2021; Welling et al. 1994).

### Spectrophotometric assays

Spectrophotometric assays are known for their user-friendly nature, requiring minimal amounts of reagents while also being applicable in high-throughput screening (Prodanović et al. 2020; Sunoqrot et al. 2021; Viña-Gonzalez et al. 2019). The assays utilize widely available laboratory equipment, such as spectrophotometers or microplate readers, which offer significant potential for scalability. This likely explains why spectrophotometric assays, encompassing colorimetric and fluorometric assays, are the most utilized assays in recent literature (SI 1). The assays rely on the detection of an increasing or diminishing chromophore or fluorophore (Eisenthal & Danson 2002). These easily detectable compounds are formed or depleted either directly due to the flavoprotein oxidase reaction or via a coupled reaction. To clarify, a chromophore is a chemical with a distinctive light absorbance feature, which, in this context, can extend outside of the visible light spectrum. A chromogen is the, often, non-detectable precursor or product of the chromophore. Similarly, fluorophores and fluorogens follow the same principle, with the distinction being that they produce a change in fluorescence signal rather than in light absorbance.

#### Colorimetric assays

The sensitivity of colorimetric assays relies on the extinction coefficient of the detected chromophore, the wavelength at which the chromophore can be monitored and, in the case of a coupled reaction, its stoichiometric relationship with the reactant. Due to advances in instruments, cuvettes, and microplates, influences of the measured wavelength is less influential for the sensitivity (Capelle et al. 2007; Perkampus 2013). Further, it is important to note that, in most cases, the extinction coefficient of the monitored chromophore needs to be experimentally determined. This is because it can vary depending on the reaction conditions, or the experiment requires the detection of a different absorbance optima than reported in literature, to avoid overlapping with absorbance of one of the other components in the reaction. The extinction coefficient can be determined using the Beer-Lambert law (1).

1 $$\mathrm{Absorbance}= \varepsilon *\text{path length}*\mathrm{concentration}$$

#### Fluorometric assays

Fluorometric assays follow the same principles as the colorimetric assay but monitor the depletion or emerging of fluorophores instead of chromophores. They are often more sensitive than colorimetric assays as the fluorescent measurements suffer from less background signal than absorbance measurements (Gul & Gribbon 2010). Its sensitivity relies on the fluorescent signal of the fluorophore and, in case of a coupled reaction, on its stoichiometric relationship with the initial reaction and its monitored wavelength. Similar to the need for determining the exact extinction coefficients of chromophores, it is essential to prepare calibration curves for the assayed fluorophore.

In short, the sensitivity of spectrophotometric assays can be mainly judged on the extinction coefficient or fluorescent yield of the followed chromophore or fluorophore and in case of a coupled reaction, its stoichiometric relationship with the flavoprotein oxidase reaction.

#### Direct spectrophotometric assay: substrates and products

Ideally, either the substrate or product of the flavoprotein oxidase-catalyzed reaction is a chromophore or fluorophore. In this way the reaction can be followed under any suitable condition for the oxidase, requiring no additional reagents, avoiding pipetting steps, and keeping the cost down. It appears to be the preferred type of activity assay as it is consistently used when available. The extinction coefficient of the monitored chromophore needs to be experimentally determined as it can vary depending on the reaction conditions. For example, vanillin is the product of several oxidase-catalyzed reactions and its formation can be monitored at 340 nm. Yet, the extinction coefficient of vanillin at this wavelength is highly pH-dependent, changing by an order of magnitude depending on the pH of the solution (García-Bofill et al. 2019; Jin et al. 2007; van den Heuvel et al. 2001). Thus, for each buffer, it is advisable to determine the exact extinction coefficient of vanillin.

For aromatic substrates or products, such as vanillin, the absorption spectrum can also vary depending on the pH (Fraaije et al. 1995). Therefore, accurate determination of the absorption maximum also needs careful consideration. This highlights the importance of determining the extinction coefficient and the absorption maximum, rather than taking it from literature. Examples of well-known flavoprotein oxidases catalyzing reactions that result in a change in absorbance due to the substrate or product being a chromophore are NAD(P)H oxidases (Koh et al. 2009), vanillyl alcohol oxidases (De Jong et al. 1992), eugenol oxidases (Jin et al. 2007), and 5-hydroxymethylfurfural oxidase (Dijkman & Fraaije 2014). In principle, also hydrogen peroxide that is typically formed by action of a flavoprotein oxidase can be monitored as chromophore as it absorbs around 240 nm (ε_240_: 40 M^−1^ cm^−1^) (Bergmeyer 1974; Bickar et al. 1982). Unfortunately, this is a relatively low value for an extinction coefficient. Two hundred forty nanometers is in a region where also other components (e.g., substrate, product, and protein) absorb light, which prohibits the use of this characteristic of hydrogen peroxide. It would also rely on a highly pure oxidase sample, devoid of catalase activity.

## Coupled reactions

Unfortunately, the majority of flavoprotein oxidase-catalyzed reactions do not inherently involve reactants that are or form chromophores or fluorophores. This prevents direct spectrophotometric activity measurements as described above. In order to monitor these reactions, an auxiliary reaction, either enzymatic or non-enzymatic, can be utilized to monitor and determine the rate of the reaction (Duggleby 1983). This can be in the form of a spontaneous reaction of a formed product with an added reactant (Gauillard et al. 1993) or via an additional (bio)catalyst and non-interfering second substrate (Bergmeyer 1965). The utilized coupled indicator reaction is preferably irreversible, sensitive and rapid to ensure precision (Easterby 1973; McClure 1969; Yang & Schulz 1987). In practical applications, this is typically achieved by incorporating an excess of indicator reactants (Cleland 1979; García-Carmona et al. 1981; Rudolph et al. 1979). Even so, a lag phase often occurs until the auxiliary reaction reaches its maximal catalytic rate (Eilertsen & Schnell 2018). Often this artifact is negligible, as the auxiliary reaction is relatively fast or, in the case of a continuous assay, one can correct for the lag phase. In a discontinuous assay this can, however, introduce an underestimation of the oxidase activity (Eisenthal & Danson 2002).

Spontaneous coupled reactions, in which a chemical reagent is used, are dependent on the product of the flavoprotein oxidase and are typically discontinuous. In literature, such assays have mainly been used for amino acid oxidases, in which the formed α-keto acid reacts with 2,4-dinitrophenylhydrazine, producing a chromophore which absorbs light at around 445 nm (Katane et al. 2020, 2007; Nagata et al. 1988). For most flavoprotein oxidases this approach is not an option because they produce different types of products. Therefore, more generic methods have been developed. These methods commonly rely on an auxiliary enzyme that reacts with the flavoprotein oxidase reaction product to either produce or deplete a chromophore or fluorophore. Among these approaches are assays utilizing an NAD(P)H-dependent enzyme, often a dehydrogenase (Gitomer & Tipton 1983). However, these are again very specific for the products of respective flavoprotein oxidase reactions. A more generic oxidase assay is the approach of using a peroxidase in combination with a flavoprotein oxidase. The peroxidase will utilize the hydrogen peroxide produced by most flavoprotein oxidases (Mattevi 2006). There is only one flavoprotein oxidase class known that does not form hydrogen peroxide as reduced dioxygen species: some NADH oxidases reduce molecular oxygen to water (Higuchi et al. 1993; Lopez de Felipe & Hugenholtz 2001; Stanton & Jensen 1993; Tanabe 1979).

### Peroxidase-based assays

**Fig. 2:**
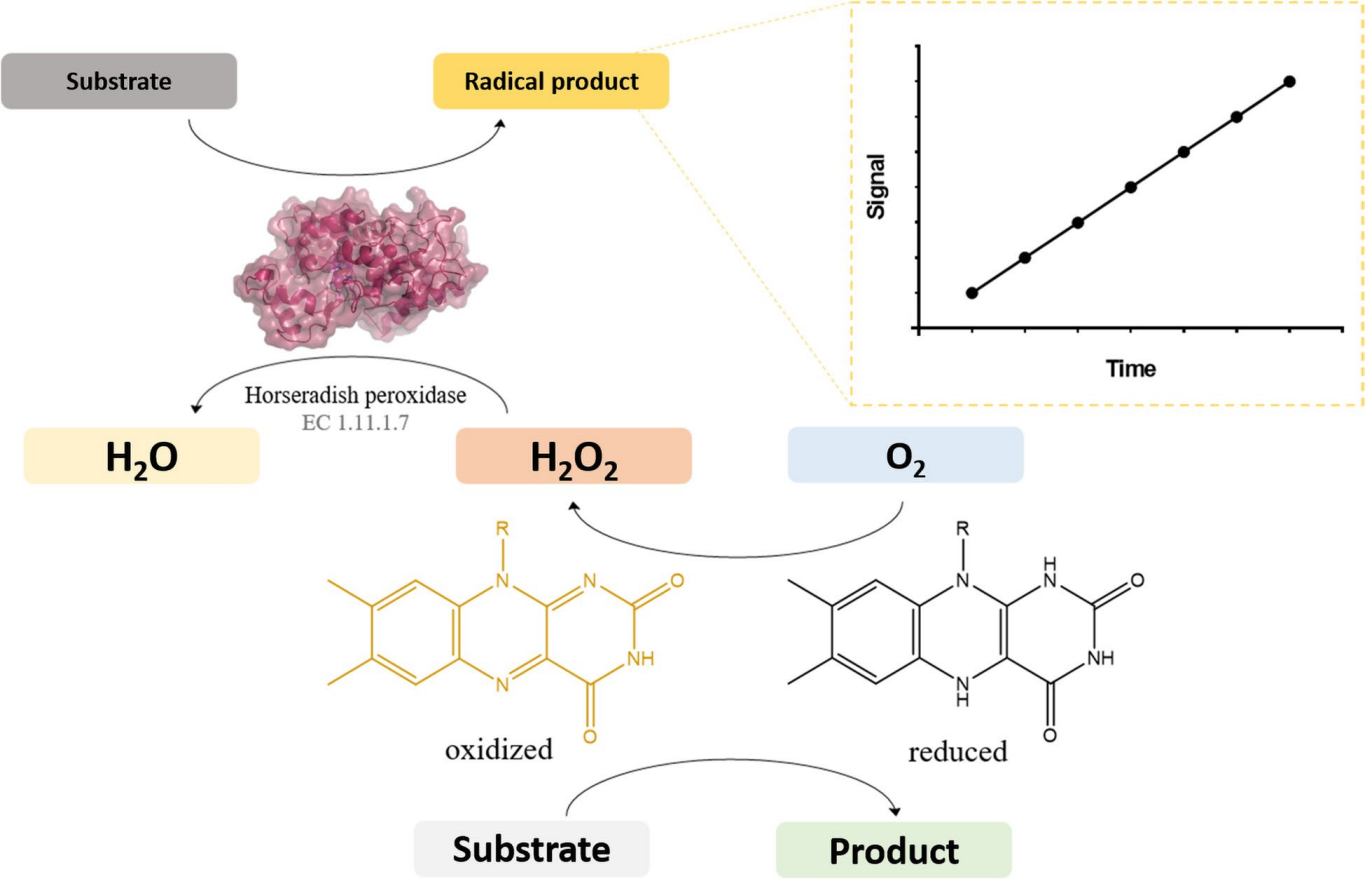
General reaction scheme of a flavoprotein oxidase, together with a coupled horseradish peroxidase reaction

Spectrophotometric assays based on an auxiliary peroxidase rely on the peroxidase-catalyzed oxidation reaction that typically results in formation of a radical as product (Veitch 2004). A number of peroxidase substrates have been identified that are suitable for spectrophotometric monitoring of such coupled reactions. The peroxidase typically generates a chromophore or fluorophore. Since these assays do not consume the product generated by the oxidase, they can even provide insight into product inhibition, although they cannot detect the rarer inhibition by hydrogen peroxide (de la Mata et al. 2000; Kleppe 1966). As mentioned above, peroxidase-based assays can be used for almost all flavoprotein oxidases relying solely on the hydrogen peroxide produced by almost all flavoprotein oxidases (Mattevi 2006) (Fig. 2). The generic character of this type of assay can simplify workflows as the same assay can be utilized for screening different flavoprotein oxidases and/or different substrates.

In literature, the peroxidase utilized in peroxidase-based assays is consistently horseradish peroxidase (HRP). This is most likely due to it having the lowest reported *K*_*M*_ (5 µM) (Gilabert et al. 2004) for hydrogen peroxide among the reported peroxidases in the BRENDA database, combined with high *k*_cat_ values for its substrates, greatly surpassing rates of flavoprotein oxidases. Flavoprotein oxidases typically exhibit *k*_cat_ values in the range of 1 to 100 s^−1^ (Mattevi 2006). The difference in turnover number between the flavoprotein oxidase and HRP, as mentioned before, is vital to follow the reaction precisely. This is also the reason why HRP and its reactants are often added in excess during the enzyme activity assays. The use of HRP as peroxidase for peroxidase-based assay may also be due to its longstanding commercial availability at a relatively low price, which goes hand-in-hand with the development of various assay methods based on HRP.

As mentioned before, this assay can be utilized for most flavoprotein oxidases, but the use of an auxiliary enzyme brings its limitations, as the working range of the assay is limited to the working range of HRP. In practical terms this implies that the assay should be performed at temperatures below 45 °C and ideally around 28 °C, in accordance with HRP’s temperature working range (Lavery et al. 2010). The pH working range of the assay is harder to define as the pH optimum for activity of HRP is slightly different depending on the used chromogen/fluorogen (Karasyova et al. 2003; Lavery et al. 2010; Munoz-Munoz et al. 2007; Nagaraja et al. 2009). HRP has been used for assays at pH 4–9, while its pH optimum for activity is around pH 6 (Schomburg et al. 2012). The broad pH range employed in assays is explained by the fact that typically an excess of HRP is used with respect to the amount of oxidase activity. This is also easily achieved due to the relatively high turnover number of HRP (200 s^−1^ at pH 6) (Violante-Mota et al. 2010).

There are variants of HRP described in literature with broader working ranges or higher turnover numbers (Cherry et al. 1999; Morawski et al. 2001) but these are thus far not utilized in activity assays due to the difficulty of expression of the HRP gene, despite recent developments in alternative expression systems (Chauhan & Kang 2018; Zhao et al. 2023). In fact, HRP is mainly offered as an enzyme purified from the roots of horseradish due to the poor expression as recombinant protein. In literature there are also alternative peroxidases, from other sources, described with broader working ranges or higher turnover number (Brissos et al. 2017; Pećanac et al. 2025; van Bloois et al. 2010), but these are thus far also not utilized in activity assays, most likely due to their obscurity or commercial unavailability.

Reactants that inhibit HRP, including thiols and heavy metals (Sariri et al. 2006; Zatón & Ochoa de Aspuru 1995; Zollner 1999), or flavoprotein oxidase substrates or products that are also accepted by HRP, such as specific phenolic compounds (Patel et al. 1997), are unsuitable for use in HRP-coupled oxidase assays. HRP is also reported to be unstable at high hydrogen peroxide concentrations (Baynton et al. 1994; Hernández-Ruiz et al. 2001; Morales-Urrea et al. 2023); these concentrations are however rarely met in activity assays as it typically only involves measurements of initial rates. Finally, the use of antioxidants, like L-ascorbic acid (Baker 1998), is not recommended with HRP-based assays as they react with the hydrogen peroxide. This results in an underestimation of oxidase activity. We also observed that commercially available HRP is not entirely pure and frequently contains trace amounts of other enzymes, among which are likely plant-derived oxidases. This underscores the importance of conducting a control experiment with the tested substrate as these contaminations can lead to false positives.

**Fig. 3:**
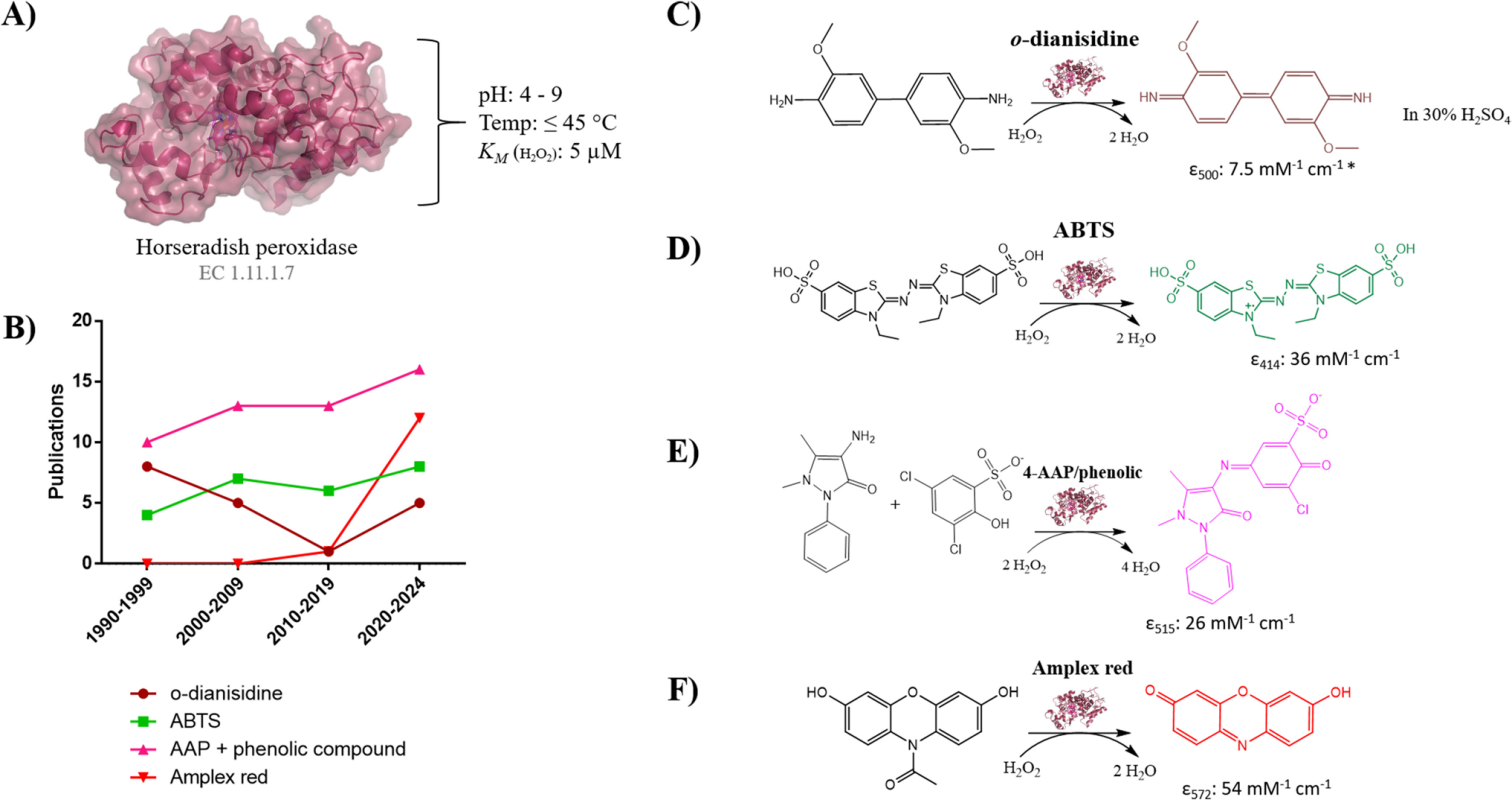
A Structure and properties of horseradish peroxidase (PDB: 1HCH). **B** Observed trend in the last 34 years in the use of different HRP-based assays for measuring flavoprotein oxidase activity (data is shown in SI 2). Amplex Red also includes Amplex UltraRed. **C** *O*-dianisidine oxidation catalyzed by HRP with hydrogen peroxide. **D** ABTS oxidization catalyzed by HRP with hydrogen peroxide.** E** 4-AAP and DCHBS oxidization catalyzed by HRP with hydrogen peroxide. **F** Amplex Red oxidization catalyzed by HRP with hydrogen peroxide

The true potential and limitations of HRP-based assays, however, are determined by the choice of chromogenic or fluorogenic substrate employed in the assay. The choice of these cosubstrates heavily influences the usability and sensitivity of the assay. An analysis of literature reporting on flavoprotein oxidases suggests that the selection of these peroxidase substrates is primarily driven by convenience, as no discernible trend could be identified (Fig. 3B, SI 2). There are a few well-established peroxidase substrates that are used in most cases. Recent literature (post 2020) shows the utilization of four popular HRP-based assays in which *o*-dianisidine, 2,2-azino-bis(2-ethylbenzthiazoline- 6-sulfonic acid) (ABTS), 4-aminoantipyrine (4-AAP) coupled with a phenolic compound or Amplex Red is utilized as chromogen or fluorogen. HRP displays high peroxidase activity (Feng et al. 2008; Glettenberg & Niemeyer 2009; Kamal & Behere 2003; Ugarova et al. 1981) for all these compounds while exhibiting a relatively low *K*_*M*_ for hydrogen peroxide. All of these assays are designed to include an excess of the chromogen/fluorogen, with their sensitivity determined by the extinction coefficient (or fluorescent yield) and the stoichiometric relationship between the resulting chromophore or fluorophore and hydrogen peroxide. Recommending an appropriate set of chromogens/fluorogens to use for an HRP-based assay is challenging as each has its own merits and a decision needs to be made based on sensitivity, cost, safety, stability of chromophore or fluorophore. To give a guideline, below, a summary is provided of the most utilized HRP-based assays. An overview of the different systems is shown in Fig. 3 and a list of chromogens/fluorogens, and their properties are given in Table 1.

**Table 1:** Commonly used chromogens and fluorogens in HRP-based assays

Chromogen(s)	*λ*_max_ (nm)	*ε* (mM^−1^ cm^−1^)	Price per 100 µL reaction (€)	Recommended use	References
*o*-Dianisidine (+ H_2_SO_4_)	436 500	8.31^a^ 7.5	0.05 × 10^−4^ (0.2 mM) 0.2 × 10^−4^ (1 mM)	Cheap and good for disrupted cell screenings Discontinuous version recommended	(Adachi et al. 2010; Geueke et al. 2007; Jimbo et al. 2003; Yang et al. 2005) (Dave et al. 2023; McComb & Yushok 1958)
*o*-Dianisidine dihydrochloride (+ H_2_SO_4_)	440 540	13^a^ 9.6	0.3 × 10^−4^ (1 mM) 0.5 × 10^−4^ (1.5 mM)	Cheap, water soluble and good for disrupted cell screenings Discontinuous version recommended	(Khan et al. 2024) (Lehmann et al. 1974; Schosinsky et al. 1974)
*o*-Tolidine (+ acid)	630^b^	*n.d*	1 × 10^−4^ (1 mM)	Not recommended	(Đurić & Deletić, 2020)
*p-*Anisidine	458	*n.d*	0.7 × 10^−4^ (1 mM)	Not recommended	(Avila & de La Guardia 1997)
*p*-Cresol	277^b^ (Decrease)	1.7^a^	0.8 × 10^−4^ (1 mM)^c^	Not recommended	(Hewson & Dunford 1976a, 1976b)
*o*-Phenylenediamine	418^b^	16.7^a^	0.5 × 10^−4^ (3.7 mM)	Not recommended	(Fornera & Walde 2010)
3,3′-Diaminobenzidine	352^b^	*n.d.*^a^	9 × 10^−4^ (0.36 mM)	Not recommended	(Cohen 1973; Sannia et al. 1991)
ABTS	414	36	5.5 × 10^−4^ (100 µM)	Water soluble, user-friendly and multiple absorbance optima	(Childs & Bardsley 1975; Wang & Reckhow 2016)
4-Chloro- 1-naphthol	550^b^	*n.d*	10 × 10^−4^ (4 mM)	Membrane staining/membrane activity screenings	(Conyers & Kidwell 1991; Ulyashova et al. 2011)
2′,7′-Dichlorodihydrofluorescein diacetate	502 (Can also be used as fluorogen)^b^	91 (Stoichiometric relationship of 5 with hydrogen peroxide)	10 × 10^−4^ (0.25 mM)	Not recommended	(Chen et al. 2010; Köchli & Von Wartburg 1978; Setini et al. 2005)
AAP/phenol	505	6.58^a^	1.7 × 10^−4^ (1.5 + 2 mM)	Not recommended	(Doukyu et al. 2008; Mortarino et al. 1996; Motoyama et al. 2022; Sugiura et al. 2021; Yamashita et al. 2000)
AAP/4-hydroxybenzoic acid	500	5.3	1.5 × 10^−4^ (1.2 + 2 mM)	Not recommended	(Iwamoto et al. 1996; Meiattini et al. 1978)
AAP/phenol- 4-sulfonic	490	5.56	4 × 10^−4^ (0.4 + 25 mM)	Not recommended	(Vojinović et al. 2004, 2007)
AAP/2,4,6-tribromo- 3-hydroxybenzoicacid	510	29.4	0.9 × 10^−4^ (0.75 + 2 mM)^c^	Appears to be in use for *L-*amino acid oxidases	(Braun et al. 1992; Trinder & Webster 1984)
AAP/DCHBS	515	26	0.3 × 10^−4^ (0.1 + 1 mM)	Water soluble, good cost, sensitive balance and produces a stable signal	(Boverio et al. 2023; Fossati et al. 1980; Tjallinks et al. 2023a)
Phenol-Red (+ NaOH)	610	*n.d*	0.4 × 10^−4^ (1 mM)	Not recommended	(Johnston et al. 2008; Pick & Keisari 1980)
***Fluorogen(s)***	*λ*_*ex*_/*λ*_*em*_ (nm)				
Scopoletin	360/465 (decrease)	*n.d*	3200 × 10^−4^ (2 mM)^c^	Not recommended	(Andreae 1955; Brotea & Thibert 1988; Corbett 1989)
Homovanillic acid	320/420	*n.d*^a^	20 × 10^−4^ (1 mM)	Cheap fluorogen option	(Faccio et al. 2010; Guilbault et al. 1967)
Luminol	351/415	Depended on solution^a^	30 × 10^−4^ (100 µM)	Not recommended	(Díaz et al. 1996; Dure & Cormier 1964)
Amplex Red	563/587	54 (572 nm)	700 × 10^−4^ (50 µM)	Extreme high sensitivity	(Mohanty et al. 1997; Zhou et al. 1997)
Amplex UltraRed	568/581	> 54^a^ (570 nm)^b^	2500 × 10^−4^ (100 µM)	Extreme high sensitivity and stability at lower pH values	(Invitrogen, 2009)

Price per reaction is based on only the price of the chromogen found at www.merck.com (08/2024). The amount of chromogen or fluorogen is based on the protocol paper added in the table together with a total reaction volume of 100 μL. Prices are taken from solid versions of chromogen or fluorogen, kit prices are not checked, and prices are calculated based on the lowest quantity that is available for purchase. ^a^Check the extinction coefficient with a calibration curve as reports are inconsistent, it is highly sensitive to the used conditions, or use in literature always is accompanied by a calibration curve. ^b^Max absorbance is either highly dependent on conditions or could not be confirmed ^c^Concentration could not be verified

### O-Dianisidine as peroxidase substrate

The development of HRP-based oxidase assays began in the mid- 1950 s as a method to allow measurements of blood glucose levels using glucose oxidase (Keston 1956). Herein, *o*-dianisidine was used as chromogen, and quickly after this publication, the assay was utilized for the characterization of oxidases (Crowne & Mansford 1962; Farmer et al. 1960; McComb et al. 1957). The assay relies on the stoichiometric 1:1 oxidation of *o*-dianisidine with hydrogen peroxide by HRP, which results in a brownish colored product with an absorbance maximum at 460 nm (Blaedel & Uhl 1975; Claiborne & Fridovich 1979a). The extinction coefficients in older literature are reported to be 30 mM^−1^ cm^−1^ at 460 nm (Lebedeva et al. 1977; Moller & Ottolenghi 1966; Rogozhin et al. 2000; Ugarova et al. 1981) and 15 mM^−1^ cm^−1^ at 450 nm (Savitsky et al. 1994). These values, however, are questionable as newer literature utilizing this assay follows formation of the product at 436 nm with a reported extinction coefficient of 8.31 mM^−1^ cm^−1^ (Adachi et al. 2010; Geueke et al. 2007; Jimbo et al. 2003; Yang et al. 2005). Still, due to inconsistency in reported extinction coefficients without proper explanations, the instability of the formed dye (Dohnal & Zyka 1974) and reported pH sensitivity (Claiborne & Fridovich 1979a), it is highly recommended to make a calibration curve for the assay under the required conditions instead of taking an extinction coefficient from literature. The working pH range of the assay is reported to be between pH 4 and pH 7 (Blecher & Glassman 1962). *O*-dianisidine is poorly soluble in water and is typically solubilized in methanol. To avoid using an organic cosolvent, the water-soluble salt, *o*-dianisidine dihydrochloride, can better be utilized. Such *o*-dianisidine solution displays an absorbance maximum at 440 nm with an extinction coefficient of 13 mM^−1^ cm^−1^ (Khan et al. 2024). The instable formed dye can be stabilized by converting the assay to a discontinues format where 30–50% H_2_SO_4_ is added after the reaction (McComb & Yushok 1958). This stabilizes the formed dye, reaching half-life values of up to 161 h (Gabler et al. 2000). The addition of an acid shifts the maximal absorbance for oxidized *o*-dianisidine to 500 nm with an extinction coefficient of 7.5 mM^−1^ cm^−1^ (Dave et al. 2023; McComb & Yushok 1958) and for *o*-dianisidine dihydrochloride to 540 nm with an extinction coefficient of 9.6 mM^−1^ cm^−1^ (Lehmann et al. 1974; Schosinsky et al. 1974). These extinction coefficients appear to be consistent in newer literature (Alapati & Handanahal 2020; Dave et al. 2023; Rayapati et al. 2024). Different acids can be used to stop the reaction and stabilize the formed chromophore, but these will slightly alter the absorbance maxima and the extinction coefficients (Porstmann et al. 1981). A more pressing limitation is that both *o*-dianisidine and its soluble analogue, although debated (Golka et al. 2004), are classified as carcinogenic (Martin & Kennelly 1981). This might explain its decline in recent years despite its low price (Table 1). The assay still finds use in screening campaigns where oxidase activity is screened for in cell extracts. In such experiments, one takes advantage of *o*-dianisidine acting as an inhibitor for catalases, native in most laboratory strains, minimizing interference caused by hydrogen peroxide consumption by these catalases (Claiborne & Fridovich 1979b; Gabler et al. 2000).

#### ABTS as peroxidase substrate

The desire to find alternative non-carcinogenic chromogens for oxidase assays led to the discovery of 2,2-azino-bis(2-ethylbenzthiazoline- 6-sulfonic acid) (ABTS) in the early 1970 s. It was described as being four times more sensitive than *o*-dianisidine, water soluble, not carcinogenic and a highly stable chromophore (t_1/2_: 48 h) (Erel 2004; Gawehn et al. 1970; Werner et al. 1970). However, it is important to note that subsequent studies revealed that ABTS is mutagenic, which asks for cautious handling of this compound (Hosoda et al. 1986). The assay relies on the one-electron oxidation of ABTS by HRP with a 2:1 stoichiometric relation with hydrogen peroxide (Cai et al. 2018; Rodríguez-López et al. 2001) to form the corresponding radical, ABTS^+^. ABTS itself can be detected at 340 nm with an extinction coefficient of 36 mM^−1^ cm^−1^ and its blue-green radical has a broad absorbance spectrum and can be followed at 414 nm with an extinction coefficient of 36 mM^−1^ cm^−1^ (Childs & Bardsley 1975), or at one of its other peaks (ε_650_: 10.2 mM^−1^ cm^−1^; ε_732_: 13.7 mM^−1^ cm^−1^; and ε_820_: 10.8 mM^−1^ cm^−1^) (Wang & Reckhow 2016). The radical is also frequently monitored at 405 nm (ε_405_: 31.6 mM^−1^ cm^−1^), enabling more precise measurements (Pinkernell et al. 1997, 2000). Having such a broad absorbance spectrum to follow the chromophore enhances the utility of the assay. For each experimental set-up, an optimal wavelength, minimizing interferences with other reaction components, can be chosen. ABTS is a more expensive chemical when compared with *o*-dianisidine (Table 1) but the price per reaction remains low. It suffers, however, from a few notable artifacts. The stability of the produced radical is highly pH-dependent; at higher pH values, the radical can react with hydrogen peroxide, regenerating ABTS (Barr & Aust 1993). Also, maintaining an excess of ABTS is crucial, as ABTS to hydrogen peroxide ratios of 0.5 can lead to overoxidation, producing ABTS^2+^, which precipitates from solution as a pale yellow color (Ilyasov et al. 2020; Kadnikova & Kostić, 2002; Majcherczyk et al. 1999). The stability of the ABTS^+^ chromophore is also influenced, although limited, by light, resulting in errors after relatively long exposure times (Liberato et al. 2018). Furthermore, commercial ABTS-HRP kits have been found to contain impurities of ABTS precursors and analogues, leading to artifacts at pH values above 8 (Zhang & Hess 2020), underscoring the importance of maintaining a proper pH for the assay. Despite these pH-dependent artifacts, the literature does not clearly define an optimal pH working range for the assay. While some sources suggest a pH range of 3–6.5 (Cano et al. 1998), other studies report successful use of the assay at higher pH values (up to pH 8) (Kamathewatta et al. 2020; Kopacz et al. 2011; Mendes et al. 2016; van Hellemond et al. 2008). The aforementioned artifacts, although important to take into account, are often relatively slow and cause minimal error in measuring initial activities. A more important limitation of the HRP-based ABTS assay is the tendency of ABTS^+^ to react with antioxidants (Arnao et al. 1996; Ilyasov et al. 2020; Re et al. 1999), phenolic compounds with electron-donating substituents (Osman et al. 2006), β,γ-unsaturated aryl ketones (Koo et al. 2025), and L-amino acids (Zheng et al. 2016). This reactivity renders the assay unusable if one of the reactants of the original reactions interacts with the ABTS radical. Clearly, negative and positive controls are vital to be able to use this assay type. The HRP-based ABTS assay continues to be widely employed (Fig. 3B) for both the characterization (Abrera et al. 2020; Kamathewatta et al. 2020; Punthong et al. 2022) and screening (Prodanović et al. 2020) of flavoprotein oxidases, likely due to its commercial availability, well-established protocols, and simplicity.

## 4-Aminoantipyrine in combination with a phenolic compound as peroxidase substrates

In 1969, Trinder proposed the use of 4-aminophenazone, later replaced by 4-aminoantipyrine (4-AAP), together with phenol as a non-carcinogenic alternative to *o*-dianisidine (Trinder 1969). Its subsequent use and popularity in glucose oxidase-coupled assays to measure blood glucose levels gave rise to the name “Trinder reagents” in medical papers (Bauminger 1974; Emmerson et al. 1973; Lott & Turner 1975). The assay gives rise to a pink quinoneimine dye which is stable, exhibits no signal loss within the first 30 min (Artiss et al. 1981), and has a stoichiometric relationship of 1:2 with hydrogen peroxide (Vojinović et al. 2004). The formed chromophore has a maximal absorbance at 505 nm and reported extinction coefficients in comparable conditions ranging from 6.58 to 13.8 mM^−1^ cm^−1^ (Doukyu et al. 2008; Mortarino et al. 1996; Motoyama et al. 2022; Sugiura et al. 2021; Yamashita et al. 2000), underscoring the necessity of determining an extinction coefficient tailored for the specific experimental conditions. In older literature, it was preferred over ABTS as it is reported to be less pH sensitive (Drozd et al. 2016) and more stable, and the formed chromophore is less reactive than the ABTS radical (Lott & Turner 1975; Pennock et al. 1973; Sharp 1972).

Due to the stability of the formed chromophore, the pH range from the assay is reported to be dependent on the pH working range of HRP, giving it a larger pH range than the aforementioned ABTS-based assay. Despite that the use of 4-AAP and phenol was initially proposed as alternative to avoid the use of carcinogenic chemicals, it was found that phenol is also toxic (Gami et al. 2014). To circumvent the use of phenol, alternatives were quickly reported that offer reduced toxicity, enhanced sensitivity, and improved water solubility (Barham & Trinder 1972; Meiattini et al. 1978; Trivedi et al. 1978; Wong et al. 1981). Notable examples still being used today are as follows: the less toxic and more water soluble phenol- 4-sulfonic acid (ε_490_: 5.56 mM^−1^ cm^−1^) (Vojinović et al. 2004, 2007), the more sensitive and less toxic 2,4,6-tribromo- 3-hydroxybenzoicacid (ε_510_: 29.4 mM^−1^ cm^−1^) (Trinder & Webster 1984) and the more sensitive, water soluble, and less toxic 3,5-dichloro- 2-hydroxybenzenesulfonic acid (DCHBS) (ε_515_: 26 mM^−1^ cm^−1^) (Fossati et al. 1980). The produced dye of the 4-AAP/DCHBS HRP-based assay (Fig. 3E) appears to be resilient as the reported extinction coefficients in literature remains consistent across various experimental conditions (Boverio et al. 2023; Callejón et al. 2015; Lim et al. 2006; Tjallinks et al. 2023a). The assay is affected by several notable artifacts, including the vulnerability of the phenolic compound to oxidative degradation during storage (Ngo & Lenhoff 1980). The majority of artifacts, however, arise from the secondary non-enzymatic reaction between the 4-AAP radical and the phenolic chromogen. Strong reducing agents, like *p*-diphenols, can react with the 4-AAP radical to produce *p*-quinones, preventing color formation (Tarasek et al. 2020) and giving rise to false negatives with the assay. Multiple different chemicals have been reported to react with the radical intermediate with no clear explanation (Genzen et al. 2016; Karon et al. 1998; Witte et al. 1978), and some chemicals are known to give rise to false positives with the assay. These potential artifacts again stress the importance of performing negative and positive controls while using the HRP/4-AAP/phenol assay. The 4-AAP-based assays remain popular (Fig. 3B) due to their low cost (Table 1), good sensitivity, stability of the produced chromophore and safety.

### Amplex Red as peroxidase substrate

When compared with chromogenic assays, fluorescence-based measurements generally show less background interference (Gul & Gribbon 2010). Accordingly, during the early stages of HRP-based assay development, several fluorophores and fluorogens were proposed (Andreae 1955; Black & Brandt 1974; Guilbault et al. 1967; Keston & Brandt 1965) and used to measure enzyme activity (Flohé & Brand 1969; Wellner & Lichtenberg 1971). One of the first developed assays was an assay involving scopoletin (6-methyl- 7-hydroxy- 1,2-benzopyrone). This aromatic compound exhibits a loss of fluorescence upon oxidation by HRP (Andreae 1955), setting it apart from the previously mentioned HRP-coupled assays, as it results in a decline in signal rather than in a gain. The stoichiometry of the reaction between scopoletin and hydrogen peroxide is 1:1, and the fluorophore can be followed by excitation at 360 nm with an emission at 465 nm (Andreae 1955; Brotea & Thibert 1988; Corbett 1989). Scopoletin is described to be stable for more than 30 min at 25 °C (Lichtenberg & Wellner 1968) but it can quickly become unstable and breaks down due to temperature and pH changes, resulting in high background signals and noise (De la Harpe & Nathan 1985; Ramasarma 1982). A solution to stabilize the remaining scopoletin signal was to quench the reaction by increasing the pH to ~ 10 (Corbett 1989), making the assay discontinuous. These complications may explain its disappearance in recent literature in favor for HRP-based assays using other fluorophores such as luminol (Díaz et al. 1996; Dure & Cormier 1964), homovanillic acid (Guilbault et al. 1967) and Amplex Red. Described first in the late 90’s (Mohanty et al. 1997; Zhou et al. 1997), to our knowledge, Amplex Red and its derivative Amplex UltraRed are the most sensitive fluorogens for HRP-based oxidase assays. HRP oxidizes Amplex Red to form resorufin, with a stoichiometric relation of 1:1 with hydrogen peroxide. Resorufin can be excited at 563 nm and emits at 587 nm or can be followed with its absorbance maxima at 572 nm with an extinction coefficient of 54 mM^−1^ cm^−1^ (Zhou et al. 1997). The stability of resorufin, which retains more than 95% of its signal after 4 h at 25 °C (Mohanty et al. 1997; Zhou et al. 1997), coupled with the high sensitivity of the assay, has established the Amplex Red assay as the gold standard for hydrogen peroxide detection. Consequently, the assay has become the benchmark for HRP-based methods in the study of flavoprotein oxidases, contributing to its increased prevalence in recent literature. Amplex Red is relatively costly compared to the assays mentioned previously (Table 1) raising the question whether the extra sensitivity compensates for the cost, as most flavoprotein oxidases can also be assayed with the less costly and less sensitive assays described above. A few notable artifacts plaguing the Amplex Red-based assay should be named. Since resorufin is a substrate of HRP (Brotea et al. 1989), albeit with turnover rates 30 times lower than those of Amplex Red, the ratio of Amplex Red to hydrogen peroxide needs to be at least five to prevent the overoxidation of resorufin (Mohanty et al. 1997; Zhou et al. 1997). Commercial Amplex Red is known to contain trace amounts of resorufin, which, in a self-catalyzing reaction with oxygen under light exposure, can create superoxide and hydrogen peroxide, which subsequently react with Amplex Red to produce more resorufin (Summers et al. 2013; Zhao et al. 2012). Since this is known to happen even by exposure to room light, the stock of Amplex Red needs to be stored under dark conditions. The signal caused by this artifact is relatively low and often overshadowed by any oxidase activity. However, when extreme sensitivity is required, the assay can be done in a discontinuous way in the dark, by measuring only an endpoint and avoiding light exposure. Further, the assay is incompatible with NAD(P)H (Zhao et al. 2011), as it reacts with resorufin. Also, the use of peroxynitrite in the assay should be avoided as HRP can use it to oxidize Amplex Red (Dębski et al. 2016). A more recent analogue of Amplex Red, known only as Amplex UltraRed, has been developed to avoid most of the previously mentioned artifacts. It exhibits reduced photosensitivity, enhanced sensitivity, and a broader pH operating range. Resorufin, the fluorescent product of the Amplex Red assay, maintains stable emission between pH 6 and pH 10 (with detectable signals as low as pH 5) and has an optimal sensitivity between pH 7.5 and 8 (Towne et al. 2004). Amplex UltroxRed, the fluorescent product of the Amplex UltraRed assay, demonstrates stable fluorescent emission from pH 5 to pH 10, with detectable signals starting at pH 4 (Invitrogen, 2009; Zhu et al. 2010). Amplex UltraRed also has a stoichiometric relation of 1:1 with hydrogen peroxide and its product Amplex UltroxRed has excitation/emission maxima of ~ 568/581 nm (Bulina et al. 2006). The extinction coefficient of Amplex UltroxRed is reported to be higher than that of resorufin and studies employing this assay typically generate calibration curves to establish the appropriate extinction coefficient or fluorescent yield under specific experimental conditions (Muellers et al. 2023). Both Amplex Red and Amplex UltraRed suffer from artifacts at high hydrogen peroxide concentrations (> 100 µM), which translates in practical terms that incubation times with the assay should be short or oxidase concentrations should be low (Towne et al. 2004). Amplex Red appears to be more used than Amplex UltraRed in papers describing flavoprotein oxidases (10:1, SI 2); this is most likely due to Amplex UltraRed being more costly than Amplex Red (Table 1) and due to its shorter time on the market. However, if a lower pH is required and the sensitivity of the Amplex Red is needed, Amplex UltraRed is the recommended fluorogen.

### Xylenol orange

**Fig. 4:**
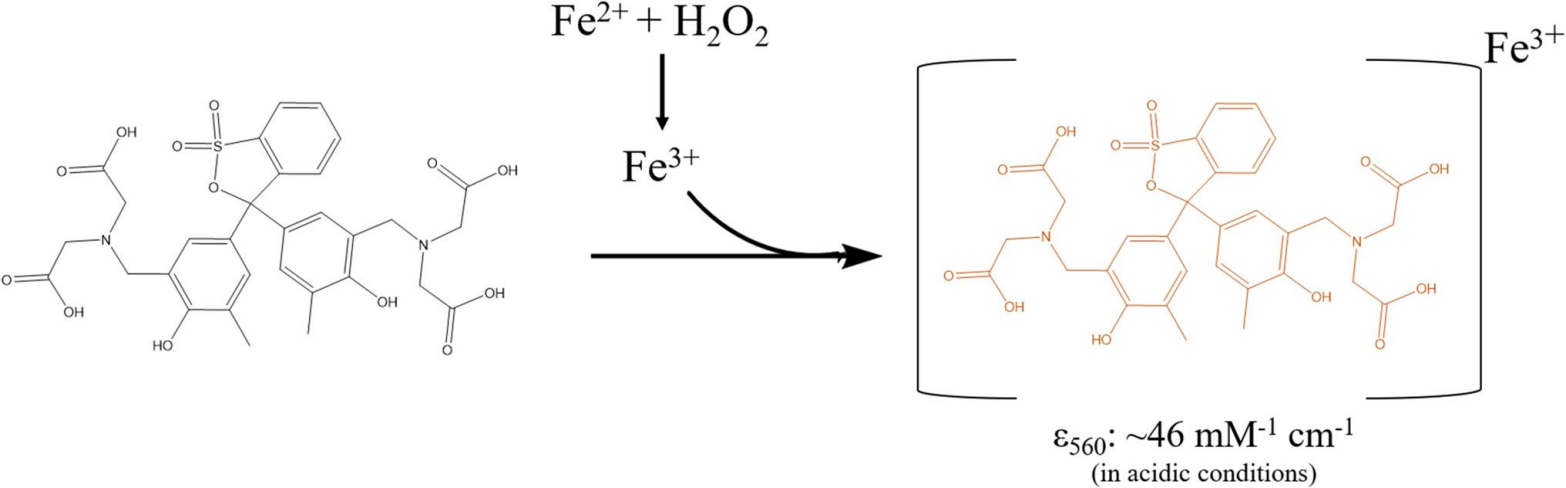
Oxidation of ferrous to ferric iron by hydrogen peroxide under acidic conditions followed by complex formation of xylenol orange with ferric iron to form a chromophore complex. The reported extinction coefficient is an estimation and is highly variable based on used experimental conditions

The hydrogen peroxide generated by flavoprotein oxidase-catalyzed reactions can also be monitored through non-enzymatic methods. By utilizing one or more redox-active compounds, similar chromogen/chromophore reactions can be catalyzed as described before. A commonly used non-enzymatic coupled assay is the xylenol orange assay (Fig. 4), in which hydrogen peroxide oxidizes the ferrous ion, producing a ferric radical which reacts with the xylenol orange dye (3,3′-bis(N,N-bis(carboxymethyl)aminomethyl)-*o*-cresolsulfonephthalein) in a ~ 1:4 stoichiometric relationship to form a chromophore complex (Gupta 1973). The reaction proceeds under highly acidic conditions, making the assay discontinuous. The resulting chromophore, which develops after 30 min, is reported to be highly stable, persisting for over 25 h and can be monitored at 560 nm (Gay et al. 1999b). However, its stability can vary depending on specific experimental conditions (Gay et al. 1999a, 1999b). The assay was initially reported in the 1970 s (Gupta 1973), although an earlier Japanese paper mentions its potential (Otomo 1965). The assay was further developed in the early 1990 s (Jiang et al. 1990) and has seen extensive use across various fields (Eymard & Genot 2003; Nourooz-Zadeh et al. 1995). Its use for measuring flavoprotein oxidase activity increased significantly following its use in the substrate specificity screening of *para*-phenol oxidases (Ewing et al. 2018). Unlike HRP-based assays, this method is suitable for use with phenolic compounds (Henriksen et al. 1999), which explains why it was chosen for studying oxidases acting on aromatic compounds. Another advantage of the discontinuous xylenol orange-based assay is that it allows for the determination of pH optima (Eggerichs et al. 2023a, 2023b). The extinction coefficient of the assay is highly dependent on the conditions, such as the acid used (Gay & Gebicki 2002), and needs to be experimentally determined for each experimental setup. It is reported to be around 43 mM^−1^ cm^−1^ (Gay et al. 1999a) and can be influenced by adding organic cosolvents (DMSO, ethanol, and methanol) (Rhee et al. 2010) or certain carbohydrates (glucose, sorbitol) (Gay & Gebicki 2000). As a result, the assay is not suited for carbohydrate oxidases since the organic substrate and/or product may affect the chromophore properties. The assay is also known to be inhibited by various substrates, and its use is reviewed in detail elsewhere (Bou et al. 2008). In brief, the assay suffers from reproducibility problems (Gay et al. 1999a) as even the source of xylenol orange can affect its extinction coefficient. The use of the assay in literature appears to be mainly for the use with *para*-phenol oxidases, such as vanillyl alcohol oxidase (Eggerichs et al. 2023a, 2023b; Ewing et al. 2018) and related flavoprotein oxidases (Frezzini et al. 2023; Viña-Gonzalez et al. 2016, 2015).

#### Electrochemical assays: the oxygen electrode

Numerous electrochemical enzyme activity methods have been reported in literature (Eisenthal & Danson 2002). One particular electrochemical assay appears to have been used for decades to monitor flavoprotein oxidase activity. Described in 1956 (Clark Jr, 1956), the Clark electrode significantly advanced the measurement of molecular oxygen in solution by simplifying the process and broadening its accessibility for widespread application. It can follow the oxygen consumption in solution of the flavoprotein oxidase-catalyzed reaction by utilizing a negatively charged platinum cathode and an Ag/AgCl reference electrode, with a thin layer of saturated KCl and a separating Teflon membrane (Charlton et al. 1963). Due to the use of a separating Teflon membrane (5–15 µm) (Friese 1980; Ultman et al. 1981), the reaction solution rarely interferes (Fork 1972) with the measurements and is reliable at different pH values and temperatures reaching 95 °C (Pouvreau et al. 2008). Coupled with the inherent oxygen consumption of all flavoprotein oxidases (Mattevi 2006), this assay stands as a true generalist in comparison to the previously described assays. The method also utilizes a stirring bar, which assures proper mixing and is beneficial for reactions using viscous solutions or reagents. The sensitivity of the electrode is influenced by various factors, such as the diameter of the cathode, the thickness of the Teflon membrane, the precision of the setup, and the age of the cathode (Eisenthal & Danson 2002). An improper setup of the electrode can result in an uneven membrane, which decreases sensitivity and accelerates electrode degradation due to its susceptibility to poisoning (Fork 1972). Clearly, it requires some experience of the user to set up the assay correctly. An improper setup or an ageing electrode can be seen by its relatively high background noise. A normal background noise of the electrode, caused by the slow consumption of oxygen by the electrode, is reported to be around 5–10 nM O_2_ (Pouvreau et al. 2008). The acceptable amount of background noise is dependent on the activity of the flavoprotein oxidase, and the method is reported to be unreliable for oxidases with *K*_*M*_ values around or below 100 nM (Lundsgaard et al. 1978; Pouvreau et al. 2008). Its response times varies from commercially available electrodes but is reported to be as low as 1 ms (Hertz & Barenholz 1973), being sufficient for flavoprotein oxidase assaying. The reason why the assay is being less utilized than spectrophotometric assays can be contributed to several factors. First, the assay requires relatively large amounts of reactants. Nowadays, reaction chambers of commercially available instruments are in the 1-mL range. The assay is not scalable to a microtiter format, rendering the assay unusable for high throughput screening. Also, a dedicated instrument is needed for such measurements. Thus, the assay is less user-friendly when compared with spectrophotometric assays. The method is mainly utilized for determining pH optima and oxygen affinity, as seen in recent work from our research group (Tjallinks et al. 2023a, 2023b). Although various alternative methods for monitoring oxygen consumption in microtiter plates have been reported (John et al. 2003; Ladner et al. 2015; Wesolowski et al. 2008), they are, to our knowledge, not frequently used for measuring flavoprotein oxidase activity.

## Concluding remarks

This review aims to provide guidelines in selecting an appropriate activity assay and to motivate the reader to critically asses the continued use of assays based solely on convenience or tradition within laboratories. Due to the versatility of flavoprotein oxidases it is nearly impossible to recommend a specific activity assay, as it needs to be decided case by case. However, a few general remarks can be made. If it is possible to directly follow the reaction, then this is generally preferred over coupled reactions as it avoids many of the artifacts mentioned in this review. It is also recommended to prefer continuous assays over discontinuous assays as it gives more insight in kinetic behavior and avoids possible hysteric effects. The choice between spectrophotometric and electrochemical assays can be made based on viscosity of the mixture, availability of the reagents/instruments and possible interferences of the assay. If reagents are not limiting, conditions require stirring, extreme pH values, high temperatures, or one of the reagents is known to interfere with either HRP or hydrogen peroxide, then the oxygen electrode is a good option. Otherwise, spectrophotometric assays should be generally preferred due to their scalability and easier workflow. When using phenolic compounds, the xylenol orange assay is a more reliable option than HRP-coupled assays since HRP is known to react with a wide range of phenolic compounds. The choice between the chromogen or fluorogen used in HRP-based assays should be decided based on cost, required sensitivity, work safety, and stability of the produced signal. *O*-Dianisidine is the cheapest option, but it is carcinogenic, suffers from many artifacts, and should preferably be used in its discontinuous form. ABTS is a well-defined and user-friendly option but should be used at slightly acidic pH values. AAP/DCHBS is cheap and continuous and produces a stable chromophore but due to its AAP radical intermediate it is known to give rise to false positives and negatives. Amplex Red is the most sensitive chromogen but comes at a high cost. When Amplex Red is preferred but the conditions of the reactions are slightly acidic, then the even more expensive Amplex UltraRed should be considered. For most continuous HRP-based assays, it is important to note that the extinction coefficient or fluorescent yield of the produced chromophore/fluorophore is often dependent on the conditions in which it forms, making it preferable to determine it experimentally for the specific required conditions. Albeit for the more stable chromogens/fluorogens, this is less vital as small deviations in the extinction coefficient often only translate in small deviations in the found *k*_*obs*_ values.

With the increasing implementation of high-throughput screening approaches, there is a growing demand for activity assays that can be performed directly in crude cell extracts or with permeabilized cells, thereby omitting enzyme purification steps. Most of the assays described in this review should be compatible with such approaches. However, such extracts often contain native catalases, peroxidases, and other compounds, which may interfere with assay outcomes. The inclusion of specific inhibitors, such as hydroxylamine (Eggerichs et al. 2024), may therefore be necessary to suppress the native consumption of hydrogen peroxide. Also, the high affinity of HRP for hydrogen peroxide may lower the risk of interfering activities. Clearly, appropriate controls are essential when setting up such high-throughput oxidase activity measurements.

With the exception of Amplex UltraRed, the assays reviewed here are relatively dated, and over the years, numerous artifacts and limitations have been identified in these methods. With the identification of novel flavoprotein oxidases, bringing new distinct reaction conditions and reagents, it is likely that additional challenges with these assays will be identified. These considerations require continuous refinement and optimization for the current assays or development of new robust assays. Promising assays may also lay hidden in literature. For example, a novel activity assay with potential for high-throughput scalability involves the use of a redox-sensitive green fluorescent protein tethered to a peroxidase which can be used to probe oxidase activity in whole cells. This methodology has been demonstrated for the identification of pyranose oxidase mutants (Herzog et al. 2020). In another study, it was shown that the H_2_O_2_-sensitive transcriptional regulator, OxyR, can be used in detecting cells that harbor oxidase activity (Kardashliev et al. 2021). Such cell-based methods allow screening of a large number of cells for oxidase activity but are not yet suitable for detailed analyses of enzyme performance.

Overall, we hope that this review encourages researchers to critically evaluate their choice of activity assays for flavoprotein oxidases or other redox enzymes, to develop new assay methodologies, and to explore underutilized or overlooked assays described in the literature.

## Supplementary Information

Below is the link to the electronic supplementary material.

Supplementary file1 (XLSX 21 KB)

Supplementary file2 (XLSX 23 KB)

## Data Availability

No datasets were generated or analysed during the current study.

## References

[CR1] Abrera AT, Chang H, Kracher D, Ludwig R, Haltrich D (2020) Characterization of pyranose oxidase variants for bioelectrocatalytic applications. Biochimica et Biophysica Acta (BBA) - Proteins and Proteomics 1868(2):140335. 10.1016/j.bbapap.2019.140335 31785381 10.1016/j.bbapap.2019.140335 PMC6949865

[CR2] Adachi MS, Torres JM, Fitzpatrick PF (2010) Mechanistic studies of the yeast polyamine oxidase fms1: kinetic mechanism, substrate specificity, and pH dependence. Biochemistry 49(49):10440–10448. 10.1021/bi1016099 21067138 10.1021/bi1016099 PMC2999662

[CR3] Alapati K, Handanahal SS (2020) Characterization of cholesterol oxidase from a marine Streptomyces sp. and its cytotoxicity. Process Biochemistry 89:175–185. 10.1016/j.procbio.2019.10.024

[CR4] Andreae WA (1955) A sensitive method for the estimation of hydrogen peroxide in biological materials. Nature 175(4463):859–860. 10.1038/175859a0 14370240 10.1038/175859a0

[CR5] Apweiler R, Armstrong R, Bairoch A, Cornish-Bowden A, Halling PJ, Hofmeyr J-HS, Kettner C, Leyh TS, Rohwer J, Schomburg D, Steinbeck C, Tipton K (2010) A large-scale protein-function database. Nat Chem Biol 6(11):785–785. 10.1038/nchembio.460 20956966 10.1038/nchembio.460 PMC3245624

[CR6] Arnao MB, Cano A, Hernández-Ruiz J, García-Cánovas F, Acosta M (1996) Inhibition byl-ascorbic acid and other antioxidants of the 2,2′-azino-bis(3-ethylbenzthiazoline-6-sulfonic acid) oxidation catalyzed by peroxidase: a new approach for determining total antioxidant status of foods. Analytical Biochemistry 236(2):255–261. 10.1006/abio.1996.0164 8660502 10.1006/abio.1996.0164

[CR7] Artiss JD, Thibert RJ, McIntosh JM, Zak B (1981) Study of various substrates for peroxidase-coupled peroxide oxidations. Microchemical Journal 26(4):487–505. 10.1016/0026-265X(81)90137-5

[CR8] Aslam, S., Ul Islam, S., Ganai, K. A., & Patro, E. R. (2020). Impact of biotechnology on the climate change. In R. M. Singh, P. Shukla, & P. Singh (Eds.), *Environmental Processes and Management: Tools and Practices* (pp. 109–120). Springer International Publishing. 10.1007/978-3-030-38152-3_7

[CR9] Atkin KE, Reiss R, Koehler V, Bailey KR, Hart S, Turkenburg JP, Turner NJ, Brzozowski AM, Grogan G (2008) The structure of monoamine oxidase from aspergillus niger provides a molecular context for improvements in activity obtained by directed evolution. Journal of Molecular Biology 384(5):1218–1231. 10.1016/j.jmb.2008.09.090 18951902 10.1016/j.jmb.2008.09.090

[CR10] Avila GP, de La Guardia M (1997) Enzymic determination of peroxides in non-aqueous media. Analyst 122(12):1543–1547

[CR11] Baker WL (1998) Removal of ascorbic acid interference in horseradish peroxidase estimations of hydrogen peroxide. Anal Lett 31(8):1325–1335. 10.1080/00032719808002869

[CR12] Barham D, Trinder P (1972) An improved colour reagent for the determination of blood glucose by the oxidase system. Analyst 97(1151):142–145. 10.1039/AN9729700142 5037807 10.1039/an9729700142

[CR13] Barr DP, Aust SD (1993) On the mechanism of peroxidase-catalyzed oxygen production. Archives of Biochemistry and Biophysics 303(2):377–382. 10.1006/abbi.1993.1298 8390221 10.1006/abbi.1993.1298

[CR14] Bauminger BB (1974) Micro method for manual analysis of true glucose in plasma without deproteinization. J Clin Pathol 27(12):1015–1017. 10.1136/jcp.27.12.1015 4452743 10.1136/jcp.27.12.1015 PMC475577

[CR15] Baynton KJ, Bewtra JK, Biswas N, Taylor KE (1994) Inactivation of horseradish peroxidase by phenol and hydrogen peroxide: a kinetic investigation. Biochimica et Biophysica Acta (BBA) - Protein Structure and Molecular Enzymology 1206(2):272–278. 10.1016/0167-4838(94)90218-6 8003531 10.1016/0167-4838(94)90218-6

[CR16] Bergmeyer H-U (1965) Methods of enzymatic analysis. Elsevier

[CR17] Bergmeyer, H. (1974). Methoden der enzymatischen analyse. Verlag Chemie. In: Weinheim.

[CR18] Bickar D, Bonaventura J, Bonaventura C (1982) Cytochrome c oxidase binding of hydrogen peroxide. Biochemistry 21(11):2661–2666 6284205 10.1021/bi00540a013

[CR19] Binoy, A., Sahadevan, R., Chaturvedi, S., & Sadhukhan, S. (2022). The pioneering role of enzymes in the valorization of waste: an insight into the mechanism of action. In P. Verma (Ed.), *Thermochemical and Catalytic Conversion Technologies for Future Biorefineries: Volume 1* (pp. 79–123). Springer Nature Singapore. 10.1007/978-981-19-4312-6_4

[CR20] Bisswanger H (2014) Enzyme assays. Perspectives in Science 1(1):41–55. 10.1016/j.pisc.2014.02.005

[CR21] Bisswanger, H. (2019). *Practical Enzymology*. Wiley. https://books.google.nl/books?id=3cecDwAAQBAJ

[CR22] Black MJ, Brandt RB (1974) Spectrofluorometric analysis of hydrogen peroxide. Analytical Biochemistry 58(1):246–254. 10.1016/0003-2697(74)90464-3 4825377 10.1016/0003-2697(74)90464-3

[CR23] Blaedel WJ, Uhl JM (1975) Nature of materials in serum that interfere in the glucose oxidase-peroxidase-0-dianisidine method for glucose, and their mode of action. Clin Chem 21(1):119–124 1116263

[CR24] Blecher M, Glassman AB (1962) Determination of glucose in the presence of sucrose using glucose oxidase; effect of pH on absorption spectrum of oxidized o-dianisidine. Analytical Biochemistry 3(4):343–352. 10.1016/0003-2697(62)90119-7 13869950 10.1016/0003-2697(62)90119-7

[CR25] Bou R, Codony R, Tres A, Decker EA, Guardiola F (2008) Determination of hydroperoxides in foods and biological samples by the ferrous oxidation–xylenol orange method: a review of the factors that influence the method’s performance. Analytical Biochemistry 377(1):1–15. 10.1016/j.ab.2008.02.029 18358821 10.1016/j.ab.2008.02.029

[CR26] Boverio A, van Beek HL, Savino S, Ranoux A, Huijgen WJJ, Raaijmakers HWC, Fraaije MW, Lončar N (2023) Biochemical and structural characterization of a uronic acid oxidase from Citrus sinensis. ChemCatChem 15(21). 10.1002/cctc.202300847

[CR27] Braun M, Kim JM, Schmid RD (1992) Purification and some properties of an extracellular l-amino acid oxidase from Cellulomonas cellulans AM8 isolated from soil. Appl Microbiol Biotechnol 37(5):594–598. 10.1007/BF00240732

[CR28] Brissos V, Tavares D, Sousa AC, Robalo MP, Martins LO (2017) Engineering a bacterial DyP-type peroxidase for enhanced oxidation of lignin-related phenolics at alkaline pH. ACS Catal 7(5):3454–3465. 10.1021/acscatal.6b03331

[CR29] Brotea GP, Draisey TF, Thibert RJ (1989) Fluorometric determination of cholesterol using an oxidase-peroxidase-resorufin system. Microchemical Journal 39(1):1–9. 10.1016/0026-265X(89)90001-5

[CR30] Brotea GP, Thibert RJ (1988) Fluorometric determination of hydrogen peroxide using resorufin and peroxidase. Microchemical Journal 37(3):368–376. 10.1016/0026-265X(88)90150-6

[CR31] Bulina ME, Chudakov DM, Britanova OV, Yanushevich YG, Staroverov DB, Chepurnykh TV, Merzlyak EM, Shkrob MA, Lukyanov S, Lukyanov KA (2006) A genetically encoded photosensitizer. Nat Biotechnol 24(1):95–99. 10.1038/nbt1175 16369538 10.1038/nbt1175

[CR32] Cai H, Liu X, Zou J, Xiao J, Yuan B, Li F, Cheng Q (2018) Multi-wavelength spectrophotometric determination of hydrogen peroxide in water with peroxidase-catalyzed oxidation of ABTS. Chemosphere 193:833–839. 10.1016/j.chemosphere.2017.11.091 29874756 10.1016/j.chemosphere.2017.11.091

[CR33] Callejón S, Sendra R, Ferrer S, Pardo I (2015) Ability of Kocuria varians LTH 1540 To Degrade Putrescine: Identification and Characterization of a Novel Amine Oxidase. J Agric Food Chem 63(16):4170–4178. 10.1021/jf5026967 25817823 10.1021/jf5026967

[CR34] Cano A, Hernández-Ruíz J, García-Cánovas F, Acosta M, Arnao MB (1998) An end-point method for estimation of the total antioxidant activity in plant material. Phytochemical Analysis: an International Journal of Plant Chemical and Biochemical Techniques 9(4):196–202

[CR35] Capelle MAH, Gurny R, Arvinte T (2007) High throughput screening of protein formulation stability: practical considerations. European Journal of Pharmaceutics and Biopharmaceutics 65(2):131–148. 10.1016/j.ejpb.2006.09.009 17107777 10.1016/j.ejpb.2006.09.009

[CR36] Chapman, J., Ismail, A. E., & Dinu, C. Z. (2018). Industrial applications of enzymes: recent advances, techniques, and outlooks. *Catalysts*,* 8*(6), 238. https://www.mdpi.com/2073-4344/8/6/238

[CR37] Charlton G, Read D, Read J (1963) Continuous intra-arterial Po2 in normal man using a flexible microelectrode. J Appl Physiol 18(6):1247–1251. 10.1152/jappl.1963.18.6.1247 14080752 10.1152/jappl.1963.18.6.1247

[CR38] Chauhan S, Kang TJ (2018) Soluble expression of horseradish peroxidase in Escherichia coli and its facile activation. Journal of Bioscience and Bioengineering 126(4):431–435. 10.1016/j.jbiosc.2018.04.004 29691194 10.1016/j.jbiosc.2018.04.004

[CR39] Chen K, Arnold FH (2020) Engineering new catalytic activities in enzymes. Nat Catal 3(3):203–213. 10.1038/s41929-019-0385-5

[CR40] Chen, K., Guo, Y., How, K., Acosta, A., Documet, D., Liang, C., Arul, D., Wood, S., Moon, K., Oliver, L. S., Fajardo, E. L., Kopyto, M., Shine, M., & Neugebauer, K. M. (2023). Five questions on how biochemistry can combat climate change. *BBA Advances*,* 4*, 100111. 10.1016/j.bbadva.2023.100111 10.1016/j.bbadva.2023.100111 PMC10709155 38075469

[CR41] Chen X, Zhong Z, Xu Z, Chen L, Wang Y (2010) 2′,7′-Dichlorodihydrofluorescein as a fluorescent probe for reactive oxygen species measurement: Forty years of application and controversy. Free Radical Res 44(6):587–604. 10.3109/10715761003709802 20370560 10.3109/10715761003709802

[CR42] Cherry JR, Lamsa MH, Schneider P, Vind J, Svendsen A, Jones A, Pedersen AH (1999) Directed evolution of a fungal peroxidase. Nat Biotechnol 17(4):379–384. 10.1038/7939 10207888 10.1038/7939

[CR43] Childs RE, Bardsley WG (1975) The steady-state kinetics of peroxidase with 2,2′-azino-di-(3-ethyl-benzthiazoline-6-sulphonic acid) as chromogen. Biochemical Journal 145(1):93–103. 10.1042/bj1450093 1191252 10.1042/bj1450093 PMC1165190

[CR44] Cipolatti, E. P., Cerqueira Pinto, M. C., Henriques, R. O., da Silva Pinto, J. C. C., de Castro, A. M., Freire, D. M. G., & Manoel, E. A. (2019). Chapter 5 - enzymes in green chemistry: the state of the art in chemical transformations. In R. S. Singh, R. R. Singhania, A. Pandey, & C. Larroche (Eds.), *Advances in Enzyme Technology* (pp. 137–151). Elsevier. 10.1016/B978-0-444-64114-4.00005-4

[CR45] Claiborne A, Fridovich I (1979a) Chemical and enzymic intermediates in the peroxidation of o-dianisidine by horseradish peroxidase 1. Spectral properties of the products of dianisidine oxidation. Biochemistry 18(11):2324–2329. 10.1021/bi00578a029 221005 10.1021/bi00578a029

[CR46] Claiborne A, Fridovich I (1979b) Purification of the o-dianisidine peroxidase from Escherichia coli B. Physicochemical characterization and analysis of its dual catalatic and peroxidatic activities. Journal of Biological Chemistry 254(10):4245–4252. 10.1016/S0021-9258(18)50722-5 374409

[CR47] Clark L Jr (1956) Monitor and control of blood and tissue oxygen tensions. ASAIO J 2(1):41–48

[CR48] Cleland WW (1979) Optimizing coupled enzyme assays. Analytical Biochemistry 99(1):142–145. 10.1016/0003-2697(79)90055-1 532954 10.1016/0003-2697(79)90055-1

[CR49] Cohen HJ (1973) The use of diaminobenzidine for spectrophotometric and acrylamide gel detection of sulfite oxidase and its applicability to hydrogen peroxide-generating enzymes. Analytical Biochemistry 53(1):208–222. 10.1016/0003-2697(73)90423-5 4145739 10.1016/0003-2697(73)90423-5

[CR50] Conyers SM, Kidwell DA (1991) Chromogenic substrates for horseradish peroxidase. Analytical Biochemistry 192(1):207–211. 10.1016/0003-2697(91)90208-B 2048722 10.1016/0003-2697(91)90208-b

[CR51] Corbett JT (1989) The scopoletin assay for hydrogen peroxide a review and a better method. Journal of Biochemical and Biophysical Methods 18(4):297–307. 10.1016/0165-022X(89)90039-0 2674266 10.1016/0165-022x(89)90039-0

[CR52] Crowne RS, Mansford KRL (1962) Studies on the specificity of commercial preparations of glucose oxidase. Analyst 87(1033):294–296. 10.1039/AN9628700294

[CR53] Dave U, Khan S, Gomes J (2023) Characterization of E121K mutation of D-amino acid oxidase – insights into mechanisms leading to amyotrophic lateral sclerosis. Biochimica et Biophysica Acta Proteins and Proteomics 1871(6):140947. 10.1016/j.bbapap.2023.140947 37558109 10.1016/j.bbapap.2023.140947

[CR54] De Jong E, Van Berkel WJH, Van Der Zwan RP, De Bont JAM (1992) Purification and characterization of vanillyl-alcohol oxidase from Penicillium simplicissimum. European Journal of Biochemistry 208(3):651–657. 10.1111/j.1432-1033.1992.tb17231.x 1396672 10.1111/j.1432-1033.1992.tb17231.x

[CR55] De la Harpe J, Nathan CF (1985) A semi-automated micro-assay for H2O2 release by human blood monocytes and mouse peritoneal macrophages. Journal of Immunological Methods 78(2):323–336. 10.1016/0022-1759(85)90089-4 3989315 10.1016/0022-1759(85)90089-4

[CR56] de la Mata I, Ramón F, Obregón V, Castillón MP, Acebal C (2000) Effect of hydrogen peroxide on d-amino acid oxidase from Rhodotorula gracilis. Enzyme and Microbial Technology 27(3):234–239. 10.1016/S0141-0229(00)00222-2 10899548 10.1016/s0141-0229(00)00222-2

[CR57] Dębski D, Smulik R, Zielonka J, Michałowski B, Jakubowska M, Dębowska K, Adamus J, Marcinek A, Kalyanaraman B, Sikora A (2016) Mechanism of oxidative conversion of Amplex® Red to resorufin: pulse radiolysis and enzymatic studies. Free Radic Biol Med 95:323–332. 10.1016/j.freeradbiomed.2016.03.027 27021961 10.1016/j.freeradbiomed.2016.03.027 PMC5697983

[CR58] Díaz AN, Sanchez FG, García JAG (1996) Hydrogen peroxide assay by using enhanced chemiluminescence of the luminol-H2O2-horseradish peroxidase system: Comparative studies. Analytica Chimica Acta 327(2):161–165. 10.1016/0003-2670(96)00077-3

[CR59] Dijkman WP, Fraaije MW (2014) Discovery and characterization of a 5-hydroxymethylfurfural oxidase from Methylovorus sp. Strain MP688. Applied and Environmental Microbiology 80(3):1082–1090. 10.1128/AEM.03740-13 24271187 10.1128/AEM.03740-13 PMC3911204

[CR60] Dohnal L, Zyka J (1974) A study of oxidation of benzidine, o, o′-tolidine, and o, o′-dianisidine. Microchemical Journal 19(1):63–70. 10.1016/0026-265X(74)90101-5

[CR61] Doukyu N, Shibata K, Ogino H, Sagermann M (2008) Purification and characterization of Chromobacterium sp. DS-1 cholesterol oxidase with thermal, organic solvent, and detergent tolerance. Applied Microbiology and Biotechnology 80(1):59–70. 10.1007/s00253-008-1526-y 18512056 10.1007/s00253-008-1526-y

[CR62] Drozd M, Pietrzak M, Parzuchowski PG, Malinowska E (2016) Pitfalls and capabilities of various hydrogen donors in evaluation of peroxidase-like activity of gold nanoparticles. Anal Bioanal Chem 408(29):8505–8513. 10.1007/s00216-016-9976-z 27722941 10.1007/s00216-016-9976-z PMC5116317

[CR63] Duggleby RG (1983) Determination of the kinetic properties of enzymes catalysing coupled reaction sequences. Biochimica et Biophysica Acta (BBA) - Protein Structure and Molecular Enzymology 744(3):249–259. 10.1016/0167-4838(83)90197-8 6849931 10.1016/0167-4838(83)90197-8

[CR64] Dure LS, Cormier MJ (1964) Studies on the Bioluminescence of Balanoglossus biminiensis Extracts: III A kinetic comparison of luminescent and nonluminescent peroxdation reactions and a proposed mechanism for peroxidase action. Journal of Biological Chemistry 239(7):2351–2359. 10.1016/S0021-9258(20)82241-8 14209968

[CR65] Đurić VR, Deletić NR (2020) Spectrophotometric determination of ascorbic acid by horseradish peroxidase. Bulletin of Natural Sciences Research 10(2):17–22

[CR66] Easterby JS (1973) Coupled enzyme assays: a general expression for the transient. Biochimica et Biophysica Acta (BBA)-Enzymology 293(2):552–558 10.1016/0005-2744(73)90362-8 4711820

[CR67] Ebrahimi Fana S, Fazaeli A, Aminian M (2023) Directed evolution of cholesterol oxidase with improved thermostability using error-prone PCR. Biotech Lett 45(9):1159–1167. 10.1007/s10529-023-03401-y 10.1007/s10529-023-03401-y 37289346

[CR68] Egbuna, C., Patrick-Iwuanyanwu, K. C., Shah, M. A., Ifemeje, J. C., & Rasul, A. (2022). Analytical Techniques in Biosciences. In C. Egbuna, K. C. Patrick-Iwuanyanwu, M. A. Shah, J. C. Ifemeje, & A. Rasul (Eds.), *Analytical Techniques in Biosciences* (pp. xv-xix). Academic Press. 10.1016/B978-0-12-822654-4.00024-5

[CR69] Eggerichs D, Weindorf N, Mascotti ML, Welzel N, Fraaije MW, Tischler D (2023a) Vanillyl alcohol oxidase from Diplodia corticola: residues Ala420 and Glu466 allow for efficient catalysis of syringyl derivatives. Journal of Biological Chemistry 299(7):104898. 10.1016/j.jbc.2023.104898 37295774 10.1016/j.jbc.2023.104898 PMC10404669

[CR70] Eggerichs D, Weindorf N, Weddeling HG, Van der Linden IM, Tischler D (2024) Substrate scope expansion of 4-phenol oxidases by rational enzyme selection and sequence-function relations. Communications Chemistry 7(1):123. 10.1038/s42004-024-01207-1 38831005 10.1038/s42004-024-01207-1 PMC11148156

[CR71] Eggerichs, D., Zilske, K., & Tischler, D. (2023b). Large scale production of vanillin using an eugenol oxidase from Nocardioides sp. YR527. *Molecular Catalysis*, *546*, 113277. 10.1016/j.mcat.2023.113277

[CR72] Eilertsen J, Schnell S (2018) A kinetic analysis of coupled (or auxiliary) enzyme reactions. Bull Math Biol 80(12):3154–3183. 10.1007/s11538-018-0513-4 30288641 10.1007/s11538-018-0513-4

[CR73] Eisenthal, R., & Danson, M. J. (2002). *Enzyme assays: a practical approach* (Vol. 257). Practical Approach.

[CR74] Emmerson AM, Garcia-Webb P, Turner SJ (1973) An antomated method for the quantitative assessment of low concentrations of glucose in urine. J Clin Pathol 26(6):454–455. 10.1136/jcp.26.6.454 4718971 10.1136/jcp.26.6.454 PMC477781

[CR75] Erel O (2004) A novel automated direct measurement method for total antioxidant capacity using a new generation, more stable ABTS radical cation. Clinical Biochemistry 37(4):277–285. 10.1016/j.clinbiochem.2003.11.015 15003729 10.1016/j.clinbiochem.2003.11.015

[CR76] Ewing, T. A., Van Noord, A., Paul, C. E., & Van Berkel, W. J. H. (2018). A xylenol orange-based screening assay for the substrate specificity of flavin-dependent para-phenol oxidases. *Molecules*, *23*(1), 164. https://www.mdpi.com/1420-3049/23/1/164 10.3390/molecules23010164 PMC6017454 29342886

[CR77] Eymard S, Genot C (2003) A modified xylenol orange method to evaluate formation of lipid hydroperoxides during storage and processing of small pelagic fish. European Journal of Lipid Science and Technology 105(9):497–501. 10.1002/ejlt.200300768

[CR78] Faccio G, Kruus K, Buchert J, Saloheimo M (2010) Secreted fungal sulfhydryl oxidases: sequence analysis and characterisation of a representative flavin-dependent enzyme from Aspergillus oryzae. BMC Biochem 11(1):31. 10.1186/1471-2091-11-31 20727152 10.1186/1471-2091-11-31 PMC2936869

[CR79] Falconer RJ, Schuur B, Mittermaier AK (2021) Applications of isothermal titration calorimetry in pure and applied research from 2016 to 2020. Journal of Molecular Recognition 34(10):e2901. 10.1002/jmr.2901 33975380 10.1002/jmr.2901

[CR80] Farmer VC, Henderson MEK, Russell JD (1960) Aromatic-alcohol-oxidase activity in the growth medium of Polystictus versicolor. Biochemical Journal 74(2):257–262. 10.1042/bj0740257 13821599 10.1042/bj0740257 PMC1204151

[CR81] Feng J-Y, Liu J-Z, Ji L-N (2008) Thermostability, solvent tolerance, catalytic activity and conformation of cofactor modified horseradish peroxidase. Biochimie 90(9):1337–1346. 10.1016/j.biochi.2008.03.010 18439429 10.1016/j.biochi.2008.03.010

[CR82] Flohé L, Brand I (1969) Kinetics of glutathione peroxidase. Biochimica et Biophysica Acta (BBA) - Enzymology 191(3):541–549. 10.1016/0005-2744(69)90347-7 10.1016/0005-2744(69)90347-7 5391643

[CR83] Fork, D. C. (1972). [10] Oxygen electrode. In *Methods in enzymology* (Vol. 24, pp. 113–122). Academic Press. 10.1016/0076-6879(72)24061-7 10.1016/0076-6879(72)24061-7 4670185

[CR84] Fornera S, Walde P (2010) Spectrophotometric quantification of horseradish peroxidase with o-phenylenediamine. Analytical Biochemistry 407(2):293–295. 10.1016/j.ab.2010.07.034 20692226 10.1016/j.ab.2010.07.034

[CR85] Fossati P, Prencipe L, Berti G (1980) Use of 3,5-dichloro-2-hydroxybenzenesulfonic acid/4-aminophenazone chromogenic system in direct enzymic assay of uric acid in serum and urine. Clin Chem 26(2):227–231. 10.1093/clinchem/26.2.227 7353268

[CR86] Fraaije MW, Veeger C, Van Berkel WJH (1995) Substrate specificity of flavin-dependent vanillyl-alcohol oxidase from Penicillium simplicissimum. European Journal of Biochemistry 234(1):271–277. 10.1111/j.1432-1033.1995.271_c.x 8529652 10.1111/j.1432-1033.1995.271_c.x

[CR87] Frezzini M, Scortica A, Capone M, Narzi D, Benedetti M, Angelucci F, Mattei B, Guidoni L (2023) Molecular dynamics simulations and kinetic measurements provide insights into the structural requirements of substrate size-dependent specificity of oligogalacturonide oxidase 1 (OGOX1). Plant Physiology and Biochemistry 194:315–325. 10.1016/j.plaphy.2022.11.021 36455304 10.1016/j.plaphy.2022.11.021

[CR88] Frieden C (1979) Slow transitions and hysteretic behavior in enzymes. Annual Review of Biochemistry 48(48):471–489. 10.1146/annurev.bi.48.070179.002351 382990 10.1146/annurev.bi.48.070179.002351

[CR89] Friese, P. (1980). A new type of Clark oxygen electrode. *Journal of Electroanalytical Chemistry and Interfacial Electrochemistry*, *106*, 409–412. 10.1016/S0022-0728(80)80187-2

[CR90] Gabler M, Hensel M, Fischer L (2000) Detection and substrate selectivity of new microbial D-amino acid oxidases. Enzyme and Microbial Technology 27(8):605–611. 10.1016/S0141-0229(00)00262-3 11024524 10.1016/s0141-0229(00)00262-3

[CR91] Gami AA, Shukor MY, Khalil KA, Dahalan FA, Khalid A, Ahmad SA (2014) Phenol and its toxicity. Journal of Environmental Microbiology and Toxicology 2(1):11–23. 10.54987/jemat.v2i1.89

[CR92] García-Bofill, M., Sutton, P. W., Guillén, M., & Álvaro, G. (2019). Enzymatic synthesis of vanillin catalysed by an eugenol oxidase. *Applied Catalysis A: General*, *582*, 117117. 10.1016/j.apcata.2019.117117

[CR93] García-Carmona F, García-Cánovas F, Lozano JA (1981) Optimizing enzyme assays with one or two coupling enzymes. Analytical Biochemistry 113(2):286–291. 10.1016/0003-2697(81)90079-8 7283134 10.1016/0003-2697(81)90079-8

[CR94] Gauillard F, Richardforget F, Nicolas J (1993) New spectrophotometric assay for polyphenol oxidase activity. Analytical Biochemistry 215(1):59–65. 10.1006/abio.1993.1554 8297016 10.1006/abio.1993.1554

[CR95] Gawehn K, Wielinger H, Werner W (1970) Screening von Chromogenen für die Blutzuckerbestimmung nach der GOD/POD-Methode. Fresenius’ Zeitschrift Für Analytische Chemie 252(2):222–224. 10.1007/BF00546390

[CR96] Gay C, Collins J, Gebicki JM (1999a) Determination of iron in solutions with the ferric–xylenol orange complex. Analytical Biochemistry 273(2):143–148. 10.1006/abio.1999.4207 10469483 10.1006/abio.1999.4207

[CR97] Gay C, Collins J, Gebicki JM (1999b) Hydroperoxide assay with the ferric–xylenol orange complex. Analytical Biochemistry 273(2):149–155. 10.1006/abio.1999.4208 10469484 10.1006/abio.1999.4208

[CR98] Gay C, Gebicki JM (2000) A critical evaluation of the effect of sorbitol on the ferric–xylenol orange hydroperoxide assay. Analytical Biochemistry 284(2):217–220. 10.1006/abio.2000.4696 10964403 10.1006/abio.2000.4696

[CR99] Gay CA, Gebicki JM (2002) Perchloric acid enhances sensitivity and reproducibility of the ferric–xylenol orange peroxide assay. Analytical Biochemistry 304(1):42–46. 10.1006/abio.2001.5566 11969187 10.1006/abio.2001.5566

[CR100] Genzen JR, Hunsaker JJ, Nelson LS, Faine BA, Krasowski MD (2016) N-acetylcysteine interference of Trinder-based assays. Clinical Biochemistry 49(1):100–104. 10.1016/j.clinbiochem.2015.10.005 26500003 10.1016/j.clinbiochem.2015.10.005

[CR101] Geueke B, Weckbecker A, Hummel W (2007) Overproduction and characterization of a recombinant D-amino acid oxidase from Arthrobacter protophormiae. Appl Microbiol Biotechnol 74(6):1240–1247. 10.1007/s00253-006-0776-9 17279391 10.1007/s00253-006-0776-9

[CR102] Gilabert MA, Fenoll LG, García-Molina F, Tudela J, García-Cánovas F, Rodríguez-López JN (2004) Kinetic characterization of phenol and aniline derivates as substrates of peroxidase. Biological Chemistry 385(9):795–800. 10.1515/BC.2004.104 15493874 10.1515/BC.2004.104

[CR103] Gitomer WL, Tipton KF (1983) The role of cytoplasmic aldehyde dehydrogenase in the metabolism of N-tele-methylhistamine. Pharmacology Biochemistry and Behavior 18:113–116. 10.1016/0091-3057(83)90156-9 6634826 10.1016/0091-3057(83)90156-9

[CR104] Glettenberg M, Niemeyer CM (2009) Tuning of peroxidase activity by covalently tethered DNA oligonucleotides. Bioconjug Chem 20(5):969–975. 10.1021/bc800558g 19334781 10.1021/bc800558g

[CR105] Golka K, Kopps S, Myslak ZW (2004) Carcinogenicity of azo colorants: influence of solubility and bioavailability. Toxicology Letters 151(1):203–210. 10.1016/j.toxlet.2003.11.016 15177655 10.1016/j.toxlet.2003.11.016

[CR106] Guilbault GG, Kramer DN, Hackley EB (1967) New substrate for fluorometric determination of oxidative enzymes. Anal Chem 39(2):271–271. 10.1021/ac60246a029 6040698 10.1021/ac60246a029

[CR107] Gul S, Gribbon P (2010) Exemplification of the challenges associated with utilising fluorescence intensity based assays in discovery. Expert Opin Drug Discov 5(7):681–690. 10.1517/17460441.2010.495748 22823207 10.1517/17460441.2010.495748

[CR108] Gupta BL (1973) Microdetermination techniques for H2O2 in irradiated solutions. Microchemical Journal 18(4):363–374. 10.1016/0026-265X(73)90059-3

[CR109] Habib MHM, Deuss PJ, Lončar N, Trajkovic M, Fraaije MW (2017) A biocatalytic one-pot approach for the preparation of lignin oligomers using an oxidase/peroxidase cascade enzyme system. Advanced Synthesis & Catalysis 359(19):3354–3361. 10.1002/adsc.201700650

[CR110] Harris T, Keshwani M (2009) Measurement of enzyme activity. Methods Enzymol 463:57–71 19892167 10.1016/S0076-6879(09)63007-X

[CR111] Henriksen A, Smith AT, Gajhede M (1999) The structures of the horseradish peroxidase c-ferulic acid complex and the ternary complex with cyanide suggest how peroxidases oxidize small phenolic substrates*. Journal of Biological Chemistry 274(49):35005–35011. 10.1074/jbc.274.49.35005 10574977 10.1074/jbc.274.49.35005

[CR112] Hernández-Ruiz J, Arnao MB, Hiner ANP, García-Cánovas F, Acosta M (2001) Catalase-like activity of horseradish peroxidase: relationship to enzyme inactivation by H2O2. Biochemical Journal 354(1):107–114. 10.1042/bj3540107 11171085 10.1042/0264-6021:3540107 PMC1221634

[CR113] Hertz R, Barenholz Y (1973) Glucose release measurements from liposomes with an oxygen electrode. Biochimica et Biophysica Acta (BBA) - Biomembranes 330(1):1–7. 10.1016/0005-2736(73)90279-4 4762768 10.1016/0005-2736(73)90279-4

[CR114] Herzog, P. L., Borghi, E., Traxlmayr, M. W., Obinger, C., Sikes, H. D., & Peterbauer, C. K. (2020). Developing a cell-bound detection system for the screening of oxidase activity using the fluorescent peroxide sensor roGFP2-Orp1. *Protein Engineering, Design and Selection*, *33*. 10.1093/protein/gzaa019 10.1093/protein/gzaa019 PMC7720637 32930800

[CR115] Hewson WD, Dunford HB (1976a) Oxidation of p-cresol by horseradish peroxidase compound I. Journal of Biological Chemistry 251(19):6036–6042. 10.1016/S0021-9258(17)33056-9 9411

[CR116] Hewson WD, Dunford HB (1976b) Stoichiometry of the reaction between horseradish peroxidase and p-cresol. Journal of Biological Chemistry 251(19):6043–6052. 10.1016/S0021-9258(17)33057-0 9412

[CR117] Higuchi M, Shimada M, Yamamoto Y, Hayashi T, Koga T, Kamio Y (1993) Identification of two distinct NADH oxidases corresponding to H2O2-forming oxidase and H2O-forming oxidase induced in Streptococcus mutans. Microbiology 139(10):2343–2351. 10.1099/00221287-139-10-2343 10.1099/00221287-139-10-2343 8254304

[CR118] Hosoda H, Takasaki W, Oe T, Tsukamoto R, Nambara T (1986) A comparison of chromogenic substrates for horseradish peroxidase as a label in steroid enzyme immunoassay. Chem Pharm Bull 34(10):4177–4182. 10.1248/cpb.34.4177 10.1248/cpb.34.4177 3829151

[CR119] Ilyasov, I. R., Beloborodov, V. L., Selivanova, I. A., & Terekhov, R. P. (2020). ABTS/PP decolorization assay of antioxidant capacity reaction pathways. *International Journal of Molecular Sciences*, *21*(3), 1131. https://www.mdpi.com/1422-0067/21/3/1131 10.3390/ijms21031131 PMC7037303 32046308

[CR120] Invitrogen, M. P. i. (2009). Amplex® UltraRed Reagent: Catalog no. A36006. In Invitrogen (Ed.).

[CR121] Iwamoto K, Suzuki K, Ikawa T (1996) Purification and characterization of glycolate oxidase from the brown alga Spatoglossum pacificism (PHAEOPHYTA) 1. J Phycol 32(5):790–798

[CR122] Jiang Z-Y, Woollard ACS, Wolff SP (1990) Hydrogen peroxide production during experimental protein glycation. FEBS Letters 268(1):69–71. 10.1016/0014-5793(90)80974-N 2384174 10.1016/0014-5793(90)80974-n

[CR123] Jimbo M, Nakanishi F, Sakai R, Muramoto K, Kamiya H (2003) Characterization of lamino acid oxidase and antimicrobial activity of aplysianin A, a sea hare-derived antitumor-antimicrobial protein. Fish Sci 69(6):1240–1246. 10.1111/j.0919-9268.2003.00751.x

[CR124] Jin J, Mazon H, van den Heuvel RHH, Janssen DB, Fraaije MW (2007) Discovery of a eugenol oxidase from Rhodococcus sp. strain RHA1. The FEBS Journal 274(9):2311–2321. 10.1111/j.1742-4658.2007.05767.x 17419730 10.1111/j.1742-4658.2007.05767.x

[CR125] John GT, Klimant I, Wittmann C, Heinzle E (2003) Integrated optical sensing of dissolved oxygen in microtiter plates: a novel tool for microbial cultivation. Biotechnology and Bioengineering 81(7):829–836. 10.1002/bit.10534 12557316 10.1002/bit.10534

[CR126] Johnston PA, Soares KM, Shinde SN, Foster CA, Shun TY, Takyi HK, Wipf P, Lazo JS (2008) Development of a 384-well colorimetric assay to quantify hydrogen peroxide generated by the redox cycling of compounds in the presence of reducing agents. Assay Drug Dev Technol 6(4):505–518. 10.1089/adt.2008.151 18699726 10.1089/adt.2008.151 PMC2752819

[CR127] Kadnikova EN, Kostić NM (2002) Oxidation of ABTS by hydrogen peroxide catalyzed by horseradish peroxidase encapsulated into sol–gel glass.: Effects of glass matrix on reactivity. Journal of Molecular Catalysis B: Enzymatic 18(1):39–48. 10.1016/S1381-1177(02)00057-7

[CR128] Kamal JKA, Behere DV (2003) Activity, stability and conformational flexibility of seed coat soybean peroxidase. Journal of Inorganic Biochemistry 94(3):236–242. 10.1016/S0162-0134(03)00004-7 12628703 10.1016/s0162-0134(03)00004-7

[CR129] Kamathewatta NJB, Deay DO III, Karaca BT, Seibold S, Nguyen TM, Tomás B, Richter ML, Berrie CL, Tamerler C (2020) Self-immobilized putrescine oxidase biocatalyst system engineered with a metal binding peptide. Langmuir 36(40):11908–11917. 10.1021/acs.langmuir.0c01986 32921059 10.1021/acs.langmuir.0c01986

[CR130] Karasyova EI, Naumchik IV, Metelitza DI (2003) Activation of peroxidase-catalyzed oxidation of aromatic amines with 2-aminothiazole and melamine. Biochem Mosc 68(1):54–62. 10.1023/A:1022193401853 10.1023/a:1022193401853 12693977

[CR131] Kardashliev T, Weingartner A, Romero E, Schwaneberg U, Fraaije M, Panke S, Held M (2021) Whole-cell screening of oxidative enzymes using genetically encoded sensors. Chem Sci 12(44):14766–14772. 10.1039/d1sc02578c 34820092 10.1039/d1sc02578c PMC8597865

[CR132] Karon BS, Daly TM, Scott MG (1998) Mechanisms of dopamine and dobutamine interference in biochemical tests that use peroxide and peroxidase to generate chromophore. Clin Chem 44(1):155–160. 10.1093/clinchem/44.1.155 9550573

[CR133] Katane M, Kuwabara H, Nakayama K, Saitoh Y, Miyamoto T, Sekine M, Homma H (2020) Biochemical characterization of d-aspartate oxidase from Caenorhabditis elegans: its potential use in the determination of free d-glutamate in biological samples. Biochimica et Biophysica Acta (BBA) - Proteins and Proteomics 1868(8):140442. 10.1016/j.bbapap.2020.140442 32376478 10.1016/j.bbapap.2020.140442

[CR134] Katane M, Seida Y, Sekine M, Furuchi T, Homma H (2007) Caenorhabditis elegans has two genes encoding functional d-aspartate oxidases. The FEBS Journal 274(1):137–149. 10.1111/j.1742-4658.2006.05571.x 17140416 10.1111/j.1742-4658.2006.05571.x

[CR135] Keston, A. (1956). Abstract of Papers, 129th Meeting. *ACS, Dallas (TX~ 310–313*.

[CR136] Keston AS, Brandt R (1965) The fluorometric analysis of ultramicro quantities of hydrogen peroxide. Analytical Biochemistry 11(1):1–5. 10.1016/0003-2697(65)90034-5 14328641 10.1016/0003-2697(65)90034-5

[CR137] Khan, S., Upadhyay, S., Dave, U., Kumar, A., & Gomes, J. (2024). Structural and mechanistic insights into ALS patient derived mutations in D-amino acid oxidase. *International Journal of Biological Macromolecules*, *256*, 128403. 10.1016/j.ijbiomac.2023.128403 10.1016/j.ijbiomac.2023.128403 38035964

[CR138] Kleppe K (1966) The effect of hydrogen peroxide on glucose oxidase from Aspergillus niger*. Biochemistry 5(1):139–143. 10.1021/bi00865a018 5938931 10.1021/bi00865a018

[CR139] Köchli H, Von Wartburg J (1978) A sensitive photometric assay for monoamine oxidase. Anal Biochem 84(1):127–135 24359 10.1016/0003-2697(78)90491-8

[CR140] Koh J-U, Chung HJ, Chang W, Tanokura M, Kong K-H (2009) Discovery and characterization of a thermostable NADH oxidase from Pyrococcus horikoshii OT3. Bull Korean Chem Soc 30:2984–2988

[CR141] Koo YS, Chen AX, Tay CYJ, Wang VYE, See JY, Lim YH, Tay DWP (2025) Navigating side reactions for robust colorimetric detection of galactose oxidase activity. Anal Chem 97(9):5266–5273. 10.1021/acs.analchem.4c07034 40021128 10.1021/acs.analchem.4c07034 PMC11912124

[CR142] Kopacz MM, Rovida S, van Duijn E, Fraaije MW, Mattevi A (2011) Structure-based redesign of cofactor binding in putrescine oxidase. Biochemistry 50(19):4209–4217. 10.1021/bi200372u 21486042 10.1021/bi200372u

[CR143] Kurbanoglu, S., Erkmen, C., & Uslu, B. (2020). Frontiers in electrochemical enzyme based biosensors for food and drug analysis. *TrAC Trends in Analytical Chemistry*, *124*, 115809. 10.1016/j.trac.2020.115809

[CR144] Ladner T, Flitsch D, Schlepütz T, Büchs J (2015) Online monitoring of dissolved oxygen tension in microtiter plates based on infrared fluorescent oxygen-sensitive nanoparticles. Microb Cell Fact 14(1):161. 10.1186/s12934-015-0347-9 26452344 10.1186/s12934-015-0347-9 PMC4600283

[CR145] Lauterbach S, Dienhart H, Range J, Malzacher S, Spöring J-D, Rother D, Pinto MF, Martins P, Lagerman CE, Bommarius AS, Høst AV, Woodley JM, Ngubane S, Kudanga T, Bergmann FT, Rohwer JM, Iglezakis D, Weidemann A, Wittig, U.,…Pleiss, J. (2023) EnzymeML: seamless data flow and modeling of enzymatic data. Nat Methods 20(3):400–402. 10.1038/s41592-022-01763-1 36759590 10.1038/s41592-022-01763-1

[CR146] Lavery CB, MacInnis MC, MacDonald MJ, Williams JB, Spencer CA, Burke AA, Irwin DJG, D’Cunha GB (2010) Purification of peroxidase from horseradish (Armoracia rusticana) roots. J Agric Food Chem 58(15):8471–8476. 10.1021/jf100786h 20681636 10.1021/jf100786h

[CR147] Lebedeva OV, Ugarova NN, Berezin IV (1977) Kinetic study of o-dianisidine oxidation by hydrogen peroxide in the presence of horseradish peroxidase. Biokhimiia 42(8):1372–1379 ((Kineticheskoe izuchenie reaktsii okisleniia o-dianizidine perekis&apos;iu vodoroda v prisutstvii peroksidazy iz khrena.)) 20989

[CR148] Lehmann HP, Schosinsky KH, Beeler MF (1974) Standardization of serum ceruloplasmin concentrations in international enzyme units with o-dianisidine dihydrochloride as substrate. Clin Chem 20(12):1564–1567. 10.1093/clinchem/20.12.1564 4430134

[CR149] Liberato A, Fernández-Trujillo MJ, Máñez Á, Maneiro M, Rodríguez-Silva L, Basallote MG (2018) Pitfalls in the ABTS peroxidase activity test: interference of photochemical processes. Inorg Chem 57(23):14471–14475. 10.1021/acs.inorgchem.8b02525 30450898 10.1021/acs.inorgchem.8b02525

[CR150] Lichtenberg LA, Wellner D (1968) A sensitive fluorometric assay for amino acid oxidases. Analytical Biochemistry 26(2):313–319. 10.1016/0003-2697(68)90343-6 4394442 10.1016/0003-2697(68)90343-6

[CR151] Lim TS, Chitra TR, Han P, Pua EC, Yu H (2006) Cloning and characterization of Arabidopsis and Brassica juncea flavin-containing amine oxidases. J Exp Bot 57(15):4155–4169. 10.1093/jxb/erl193 17122409 10.1093/jxb/erl193

[CR152] Lopez de Felipe F, Hugenholtz J (2001) Purification and characterisation of the water forming NADH-oxidase from Lactococcus lactis. International Dairy Journal 11(1):37–44. 10.1016/S0958-6946(01)00031-0

[CR153] Lott JA, Turner K (1975) Evaluation of Trinder’s glucose oxidase method for measuring glucose in serum and urine. Clin Chem 21(12):1754–1760. 10.1093/clinchem/21.12.1754 1237363

[CR154] Lundsgaard JS, Grønlund J, Degn H (1978) Error in oxygen measurements in open systems owing to oxygen consumption in unstirred layer. Biotechnology and Bioengineering 20(6):809–819. 10.1002/bit.260200604

[CR155] Majcherczyk A, Johannes C, Hüttermann A (1999) Oxidation of aromatic alcohols by laccase from Trametes versicolor mediated by the 2,2′-azino-bis-(3-ethylbenzothiazoline-6-sulphonic acid) cation radical and dication. Appl Microbiol Biotechnol 51(2):267–276. 10.1007/s002530051392

[CR156] Mandpe P, Prabhakar B, Gupta H, Shende P (2020) Glucose oxidase-based biosensor for glucose detection from biological fluids. Sens Rev 40(4):497–511

[CR157] Martin, C., Binda, C., Fraaije, M. W., & Mattevi, A. (2020). Chapter three - the multipurpose family of flavoprotein oxidases. In P. Chaiyen & F. Tamanoi (Eds.), *The Enzymes* (Vol. 47, pp. 63–86). Academic Press. 10.1016/bs.enz.2020.05.002 10.1016/bs.enz.2020.05.002 32951835

[CR158] Martin CN, Kennelly JC (1981) Rat liver microsomal azoreductase activity on four azo dyes derived from benzidine, 3,3’-dimethoxybenzidine or 3,3’-dimethoxybenzidine. Carcinogenesis 2(4):307–312. 10.1093/carcin/2.4.307 7023728 10.1093/carcin/2.4.307

[CR159] Mattevi A (2006) To be or not to be an oxidase: challenging the oxygen reactivity of flavoenzymes. Trends Biochem Sci 31(5):276–283 16600599 10.1016/j.tibs.2006.03.003

[CR160] McClure WR (1969) Kinetic analysis of coupled enzyme assays. Biochemistry 8(7):2782–2786. 10.1021/bi00835a014 4241273 10.1021/bi00835a014

[CR161] McComb RB, Yushok WD (1958) Colorimetric estimation of d-glucose and 2-deoxy-d-glucose with glucose oxidase. Journal of the Franklin Institute 265(5):417–422. 10.1016/0016-0032(58)90724-5

[CR162] McComb RB, Yushok WD, Batt WG (1957) 2-Deoxy-D-glucose, a new substrate for glucose oxidase (glucose aerodehydrogenase). Journal of the Franklin Institute 263(2):161–165. 10.1016/0016-0032(57)90762-7

[CR163] Meiattini F, Prencipe L, Bardelli F, Giannini G, Tarli P (1978) The 4-hydroxybenzoate/4-aminophenazone chromogenic system used in the enzymic determination of serum cholesterol. Clin Chem 24(12):2161–2165 719864

[CR164] Mendes, S., Banha, C., Madeira, J., Santos, D., Miranda, V., Manzanera, M., Ventura, M. R., van Berkel, W. J. H., & Martins, L. O. (2016). Characterization of a bacterial pyranose 2-oxidase from Arthrobacter siccitolerans. *Journal of Molecular Catalysis B: Enzymatic*, *133*, S34-S43. 10.1016/j.molcatb.2016.11.005

[CR165] Mohanty JG, Jaffe JS, Schulman ES, Raible DG (1997) A highly sensitive fluorescent micro-assay of H2O2 release from activated human leukocytes using a dihydroxyphenoxazine derivative. Journal of Immunological Methods 202(2):133–141. 10.1016/S0022-1759(96)00244-X 9107302 10.1016/s0022-1759(96)00244-x

[CR166] Moller KM, Ottolenghi P (1966) The oxidation of o-dianisidine by H2O2 and peroxidase at neutral pH. C R Trav Lab Carlsberg 35(16):369–389 5956185

[CR167] Morales-Urrea D, López-Córdoba A, Contreras EM (2023) Inactivation kinetics of horseradish peroxidase (HRP) by hydrogen peroxide. Sci Rep 13(1):13363. 10.1038/s41598-023-39687-1 37591893 10.1038/s41598-023-39687-1 PMC10435507

[CR168] Morawski B, Quan S, Arnold FH (2001) Functional expression and stabilization of horseradish peroxidase by directed evolution in Saccharomyces cerevisiae. Biotechnology and Bioengineering 76(2):99–107. 10.1002/bit.1149 11505379 10.1002/bit.1149

[CR169] Mortarino M, Negri A, Tedeschi G, Simonic T, Duga S, Gassen HG, Ronchi S (1996) L-Aspartate oxidase from Escherichia coli. European Journal of Biochemistry 239(2):418–426. 10.1111/j.1432-1033.1996.0418u.x 8706749 10.1111/j.1432-1033.1996.0418u.x

[CR170] Motoyama T, Yamamoto Y, Ishida C, Hasebe F, Kawamura Y, Shigeta Y, Ito S, Nakano S (2022) Reaction mechanism of ancestral l-Lys α-oxidase from caulobacter species studied by biochemical, structural, and computational analysis. ACS Omega 7(48):44407–44419. 10.1021/acsomega.2c06334 36506213 10.1021/acsomega.2c06334 PMC9730747

[CR171] Muellers SN, Tararina MA, Kuzmanovic U, Galagan JE, Allen KN (2023) Structural insights into the substrate range of a bacterial monoamine oxidase. Biochemistry 62(3):851–862. 10.1021/acs.biochem.2c00540 36662673 10.1021/acs.biochem.2c00540

[CR172] Munoz-Munoz JL, Garcia-Molina F, Varon R, Rodriguez-Lopez JN, Garcia-Canovas F, Tudela J (2007) Kinetic characterization of the oxidation of esculetin by polyphenol oxidase and peroxidase. Biosci Biotechnol Biochem 71(2):390–396. 10.1271/bbb.60431 17284853 10.1271/bbb.60431

[CR173] Nagaraja P, Shivakumar A, Kumar Shrestha A (2009) Development and evaluation of kinetic spectrophotometric assays for horseradish Peroxidase by Catalytic Coupling of Paraphenylenediamine and mequinol. Anal Sci 25(10):1243–1248. 10.2116/analsci.25.1243 19822971 10.2116/analsci.25.1243

[CR174] Nagata Y, Shimojo T, Akino T (1988) Two spectrophotometric assays for D-amino acid oxidase: for the study of distribution patterns. Int J Biochem 20(11):1235–1238. 10.1016/0020-711x(88)90225-x 2907883 10.1016/0020-711x(88)90225-x

[CR175] Ngo TT, Lenhoff HM (1980) A sensitive and versatile chromogenic assay for peroxidase and peroxidase-coupled reactions. Analytical Biochemistry 105(1):389–397. 10.1016/0003-2697(80)90475-3 7457843 10.1016/0003-2697(80)90475-3

[CR176] Nourooz-Zadeh J, Tajaddini-Sarmadi J, Wolff SP (1995) Measurement of hydroperoxides in edible oils using the ferrous oxidation in xylenol orange assay. J Agric Food Chem 43(1):17–21. 10.1021/jf00049a005

[CR177] Osman AM, Wong KK, Fernyhough A (2006) ABTS radical-driven oxidation of polyphenols: isolation and structural elucidation of covalent adducts. Biochem Biophys Res Commun 346(1):321–329. 10.1016/j.bbrc.2006.05.118 16756947 10.1016/j.bbrc.2006.05.118

[CR178] Otomo M (1965) Composition of the xylenol orange complexes of iron (III) and their application to the determination of iron or xylenol orange. Bunseki Kagaku 14(8):677–682. 10.2116/bunsekikagaku.14.677

[CR179] Patel PK, Mondal MS, Modi S, Behere DV (1997) Kinetic studies on the oxidation of phenols by the horseradish peroxidase compound II. Biochimica et Biophysica Acta (BBA) -Protein Structure and Molecular Enzymology 1339(1):79–87. 10.1016/S0167-4838(96)00219-1 9165102 10.1016/s0167-4838(96)00219-1

[CR180] Pećanac O, Martin C, Savino S, Rozeboom HJ, Fraaije MW, Lončar N (2025) Biochemical and structural characterisation of a bacterial lactoperoxidase. ChemBioChem 26(2):e202400713. 10.1002/cbic.202400713 39570012 10.1002/cbic.202400713 PMC11776367

[CR181] Pennock CA, Murphy D, Sellers J, Longdon KJ (1973) A comparison of autoanalyser methods for the estimation of glucose in blood. Clinica Chimica Acta 48(2):193–201. 10.1016/0009-8981(73)90365-3 10.1016/0009-8981(73)90365-3 4758882

[CR182] Perkampus, H.-H. (2013). *UV-VIS Spectroscopy and its Applications*. Springer Science & Business Media.

[CR183] Pick E, Keisari Y (1980) A simple colorimetric method for the measurement of hydrogen peroxide produced by cells in culture. Journal of Immunological Methods 38(1):161–170. 10.1016/0022-1759(80)90340-3 6778929 10.1016/0022-1759(80)90340-3

[CR184] Pimviriyakul, P., & Chaiyen, P. (2020). Chapter One - Overview of flavin-dependent enzymes. In P. Chaiyen & F. Tamanoi (Eds.), *The Enzymes* (Vol. 47, pp. 1–36). Academic Press. 10.1016/bs.enz.2020.06.006 10.1016/bs.enz.2020.06.006 32951820

[CR185] Pinkernell U, Lüke H-J, Karst U (1997) Selective photometric determination of peroxycarboxylic acids in the presence of hydrogen peroxide. Analyst 122(6):567–571. 10.1039/A700509A

[CR186] Pinkernell U, Nowack B, Gallard H, von Gunten U (2000) Methods for the photometric determination of reactive bromine and chlorine species with ABTS. Water Research 34(18):4343–4350. 10.1016/S0043-1354(00)00216-5

[CR187] Pleiss J (2021) Standardized Data, Scalable Documentation, Sustainable Storage – EnzymeML As A Basis For FAIR Data Management In Biocatalysis. ChemCatChem 13(18):3909–3913. 10.1002/cctc.202100822

[CR188] Porstmann B, Porstmann T, Nugel E (1981) Comparison of chromogens for the determination of horseradish peroxidase as a marker in enzyme immunoassay. Clinical Chemistry and Laboratory Medicine (CCLM) 19(7):435–440. 10.1515/cclm.1981.19.7.435 10.1515/cclm.1981.19.7.435 7035603

[CR189] Pouvreau, L. A. M., Strampraad, M. J. F., Berloo, S. V., Kattenberg, J. H., & de Vries, S. (2008). Chapter six - NO, N2O, and O2 reaction kinetics: scope and limitations of the clark electrode. In R. K. Poole (Ed.), *Methods in enzymology* (Vol. 436, pp. 97–112). Academic Press. 10.1016/S0076-6879(08)36006-6 10.1016/S0076-6879(08)36006-6 18237629

[CR190] Prodanović, R., Ung, W. L., Ilić Đurđić, K., Fischer, R., Weitz, D. A., & Ostafe, R. (2020). A high-throughput screening system based on droplet microfluidics for glucose oxidase gene libraries. *Molecules*, *25*(10), 2418. https://www.mdpi.com/1420-3049/25/10/2418 10.3390/molecules25102418 PMC7287683 32455903

[CR191] Punthong, P., Visitsatthawong, S., Chuaboon, L., Chaiyen, P., & Wongnate, T. (2022). Chemo-enzymatic synthesis of sugar acid by pyranose 2-oxidase. *Molecular Catalysis*, *533*, 112753. 10.1016/j.mcat.2022.112753

[CR192] Ramasarma T (1982) Generation of H2O2 in biomembranes. Biochimica et Biophysica Acta (BBA) - Reviews on Biomembranes 694(1):69–93. 10.1016/0304-4157(82)90014-4 6751395 10.1016/0304-4157(82)90014-4

[CR193] Range J, Halupczok C, Lohmann J, Swainston N, Kettner C, Bergmann FT, Weidemann A, Wittig U, Schnell S, Pleiss J (2022) EnzymeML—a data exchange format for biocatalysis and enzymology. The FEBS Journal 289(19):5864–5874. 10.1111/febs.16318 34890097 10.1111/febs.16318

[CR194] Rayapati AM, Vemulapati B, Chanda C (2024) Cobra (Naja naja) venom L-amino acid oxidase (NNLAAO70) induces apoptosis and secondary necrosis in human lung epithelial cancer cells. J Biosci 49(2):43. 10.1007/s12038-024-00429-8 38516910

[CR195] Re R, Pellegrini N, Proteggente A, Pannala A, Yang M, Rice-Evans C (1999) Antioxidant activity applying an improved ABTS radical cation decolorization assay. Free Radical Biology and Medicine 26(9):1231–1237. 10.1016/S0891-5849(98)00315-3 10381194 10.1016/s0891-5849(98)00315-3

[CR196] Reis, J., & Binda, C. (2023). The peroxidase-coupled assay to measure MAO enzymatic activity. In C. Binda (Ed.), *Monoamine Oxidase: Methods and Protocols* (pp. 23–34). Springer US. 10.1007/978-1-0716-2643-6_3 10.1007/978-1-0716-2643-6_3 36169853

[CR197] Rembeza E, Boverio A, Fraaije MW, Engqvist MKM (2022) Discovery of two novel oxidases using a high-throughput activity screen. ChemBioChem 23(2):e202100510. 10.1002/cbic.202100510 34709726 10.1002/cbic.202100510 PMC9299179

[CR198] Rhee SG, Chang T-S, Jeong W, Kang D (2010) Methods for detection and measurement of hydrogen peroxide inside and outside of cells. Mol Cells 29(6):539–549. 10.1007/s10059-010-0082-3 20526816 10.1007/s10059-010-0082-3

[CR199] Robinson PK (2015) Enzymes: principles and biotechnological applications. Essays Biochem 59:1–41. 10.1042/bse0590001 26504249 10.1042/bse0590001 PMC4692135

[CR200] Rodríguez-López JN, Lowe DJ, Hernández-Ruiz J, Hiner ANP, García-Cánovas F, Thorneley RNF (2001) Mechanism of reaction of hydrogen peroxide with horseradish peroxidase: identification of intermediates in the catalytic cycle. J Am Chem Soc 123(48):11838–11847. 10.1021/ja011853+ 11724589 10.1021/ja011853+

[CR201] Rogozhin VV, Kutuzova GD, Ugarova NN (2000) Inhibition of horseradish peroxidase byN-ethylamide ofo-sulfobenzoylacetic acid. Russ J Bioorg Chem 26(2):138–141. 10.1007/BF02759160 10808412

[CR202] Rosini, E., Caldinelli, L., & Piubelli, L. (2018). Assays of D-amino acid oxidase activity [Protocols]. *Frontiers in Molecular Biosciences*,* 4*. 10.3389/fmolb.2017.00102 10.3389/fmolb.2017.00102 PMC5785730 29404340

[CR203] Rudolph, F. B., Baugher, B. W., & Beissner, R. S. (1979). [2] Techniques in coupled enzyme assays. In *Methods in enzymology* (Vol. 63, pp. 22–42). Academic Press. 10.1016/0076-6879(79)63004-5 10.1016/0076-6879(79)63004-5 228153

[CR204] Sannia G, Limongi P, Cocca E, Buonocore F, Nitti G, Giardina P (1991) Purification and characterization of a veratryl alcohol oxidase enzyme from the lignin degrading basidiomycete Pleurotus ostreatus. Biochimica et Biophysica Acta (BBA) - General Subjects 1073(1):114–119. 10.1016/0304-4165(91)90190-R 1991127 10.1016/0304-4165(91)90190-r

[CR205] Sariri R, Sajedi RH, Jafarian V (2006) Inhibition of horseradish peroxidase activity by thiol type inhibitors. Journal of Molecular Liquids 123(1):20–23. 10.1016/j.molliq.2005.05.004

[CR206] Savitsky AP, Nelen MI, Yatsmirsky AK, Demcheva MV, Ponomarev GV, Sinikov IV (1994) Kinetics of oxidation of o-dianisidine by hydrogen peroxide in the presence of antibody complexes of iron(III) coproporphyrin. Appl Biochem Biotechnol 47(2):317–327. 10.1007/BF02787943 7944346 10.1007/BF02787943

[CR207] Schomburg, D., Salzmann, M., & Stephan, D. (2012). *Enzyme Handbook 7: Class 1.5–1.12: Oxidoreductases*. Springer Science & Business Media.

[CR208] Schomburg I, Chang A, Hofmann O, Ebeling C, Ehrentreich F, Schomburg D (2002) BRENDA: a resource for enzyme data and metabolic information. Trends in Biochemical Sciences 27(1):54–56. 10.1016/S0968-0004(01)02027-8 11796225 10.1016/s0968-0004(01)02027-8

[CR209] Schosinsky KH, Lehmann HP, Beeler MF (1974) Measurement of ceruloplasmin from its oxidase activity in serum by use of o-dianisidine dihydrochloride. Clin Chem 20(12):1556–1563. 10.1093/clinchem/20.12.1556 4214636

[CR210] Setini A, Pierucci F, Senatori O, Nicotra A (2005) Molecular characterization of monoamine oxidase in zebrafish (Danio rerio). Comparative Biochemistry and Physiology Part B: Biochemistry and Molecular Biology 140(1):153–161. 10.1016/j.cbpc.2004.10.002 10.1016/j.cbpc.2004.10.002 15621520

[CR211] Sharp P (1972) Interference in glucose oxidase-peroxidase blood glucose methods. Clinica Chimica Acta 40(1):115–120. 10.1016/0009-8981(72)90257-4 10.1016/0009-8981(72)90257-4 5056619

[CR212] Sindhu, R., Shiburaj, S., Sabu, A., Fernandes, P., Singhal, R., Mathew, G. M., Nair, I. C., Jayachandran, K., Vidya, J., & de Souza Vandenberghe, L. P. (2020). Enzyme technology in food processing: recent developments and future prospects. *Innovative food processing technologies: a comprehensive review*, 191–215.

[CR213] Smith L, Camerino PW (1963) Comparison of polarographic and spectrophotometric assays for cytochrome c oxidase activity*. Biochemistry 2(6):1428–1432. 10.1021/bi00906a039 14093921 10.1021/bi00906a039

[CR214] Stanton TB, Jensen NS (1993) Purification and characterization of NADH oxidase from Serpulina (Treponema) hyodysenteriae. Journal of Bacteriology 175(10):2980–2987. 10.1128/jb.175.10.2980-2987.1993 8491717 10.1128/jb.175.10.2980-2987.1993 PMC204616

[CR215] Sugiura S, Nakano S, Niwa M, Hasebe F, Matsui D, Ito S (2021) Catalytic mechanism of ancestral L-lysine oxidase assigned by sequence data mining. Journal of Biological Chemistry 297(3). 10.1016/j.jbc.2021.101043 10.1016/j.jbc.2021.101043 PMC8405998 34358565

[CR216] Summers, F. A., Zhao, B., Ganini, D., & Mason, R. P. (2013). Chapter one - photooxidation of Amplex Red to resorufin: implications of exposing the Amplex Red assay to light. In E. Cadenas & L. Packer (Eds.), *Methods in enzymology* (Vol. 526, pp. 1–17). Academic Press. 10.1016/B978-0-12-405883-5.00001-6 10.1016/B978-0-12-405883-5.00001-6 23791091

[CR217] Sunoqrot S, Al-Hadid A, Manasrah A, Khnouf R, Hasan Ibrahim L (2021) Immobilization of glucose oxidase on bioinspired polyphenol coatings as a high-throughput glucose assay platform. RSC Advances 11(62):39582–39592. 10.1039/D1RA07467A 35492494 10.1039/d1ra07467a PMC9044463

[CR218] Tanabe M (1979) Trichomonas vaginalis: NADH oxidase activity. Experimental Parasitology 48(1):135–143. 10.1016/0014-4894(79)90063-8 37100 10.1016/0014-4894(79)90063-8

[CR219] Tarasek, D., Gąsowska-Bajger, B., & Wojtasek, H. (2020). Mechanisms of interference of p-diphenols with the Trinder reaction. *Bioorganic Chemistry*, *97*, 103692. 10.1016/j.bioorg.2020.103692 10.1016/j.bioorg.2020.103692 32155504

[CR220] Tjallinks G, Boverio A, Jager AW, Kaya SG, Mattevi A, Fraaije MW (2023a) Efficient oxidation of 5-hydroxymethylfurfural using a flavoprotein oxidase from the honeybee Apis mellifera. ChemBioChem 24(24):e202300588. 10.1002/cbic.202300588 37800383 10.1002/cbic.202300588

[CR221] Tjallinks G, Boverio A, Maric I, Rozeboom HJ, Arentshorst M, Visser J, Ram AFJ, Mattevi A, Fraaije MW (2023b) Structure elucidation and characterization of patulin synthase, insights into the formation of a fungal mycotoxin. The FEBS Journal 290(21):5114–5126. 10.1111/febs.16804 37366079 10.1111/febs.16804

[CR222] Towne V, Will M, Oswald B, Zhao Q (2004) Complexities in horseradish peroxidase-catalyzed oxidation of dihydroxyphenoxazine derivatives: appropriate ranges for pH values and hydrogen peroxide concentrations in quantitative analysis. Analytical Biochemistry 334(2):290–296. 10.1016/j.ab.2004.07.037 15494136 10.1016/j.ab.2004.07.037

[CR223] Trinder P (1969) Determination of glucose in blood using glucose oxidase with an alternative oxygen acceptor. Ann Clin Biochem 6(1):24–27. 10.1177/000456326900600108

[CR224] Trinder P, Webster D (1984) Determination of HDL-cholesterol using 2,4,6-tribromo-3-hydroxybenzoic acid with a commercial CHOD—PAP reagent. Ann Clin Biochem 21(5):430–433. 10.1177/000456328402100516 6508215 10.1177/000456328402100516

[CR225] Trivedi RC, Rebar L, Berta E, Stong L (1978) New enzymatic method for serum uric acid at 500 nm. Clin Chem 24(11):1908–1911 709818

[CR226] Ugarova NN, Lebedeva OV, Berezin IV (1981) Horseradish peroxidase catalysis I. Steady-state kinetics of peroxidase-catalyzed individual and co-oxidation of potassium ferrocyanide and o-dianisidine by hydrogen peroxide. Journal of Molecular Catalysis 13(2):215–225. 10.1016/0304-5102(81)85022-5

[CR227] Ultman JS, Firouztale E, Skerpon MJ (1981) A spherical model of the clark electrode. Journal of Electroanalytical Chemistry and Interfacial Electrochemistry 127(1):59–66. 10.1016/S0022-0728(81)80467-6

[CR228] Ulyashova MM, Rubtsova MY, Egorov AM (2011) Colorimetric detection of immobilized horseradish peroxidase based on the co-oxidation of benzidine derivatives and 4-chloro-1-naphthol. Russ Chem Bull 60(4):656–661. 10.1007/s11172-011-0101-3

[CR229] van Bloois E, Torres Pazmiño DE, Winter RT, Fraaije MW (2010) A robust and extracellular heme-containing peroxidase from Thermobifida fusca as prototype of a bacterial peroxidase superfamily. Appl Microbiol Biotechnol 86(5):1419–1430. 10.1007/s00253-009-2369-x 19967355 10.1007/s00253-009-2369-x PMC2854361

[CR230] van den Heuvel RHH, Fraaije MW, Laane C, van Berkel WJH (2001) Enzymatic Synthesis of Vanillin. J Agric Food Chem 49(6):2954–2958. 10.1021/jf010093j 11409992 10.1021/jf010093j

[CR231] van Hellemond EW, van Dijk M, Heuts DPHM, Janssen DB, Fraaije MW (2008) Discovery and characterization of a putrescine oxidase from Rhodococcus erythropolis NCIMB 11540. Appl Microbiol Biotechnol 78(3):455–463. 10.1007/s00253-007-1310-4 18183391 10.1007/s00253-007-1310-4 PMC2243256

[CR232] Veitch NC (2004) Horseradish peroxidase: a modern view of a classic enzyme. Phytochemistry 65(3):249–259. 10.1016/j.phytochem.2003.10.022 14751298 10.1016/j.phytochem.2003.10.022

[CR233] Viña-Gonzalez, J., Gonzalez-Perez, D., & Alcalde, M. (2016). Directed evolution method in Saccharomyces cerevisiae: mutant library creation and screening. *JoVE*(110), e53761. 10.3791/53761 10.3791/53761 PMC4841332 27077451

[CR234] Viña-Gonzalez J, Gonzalez-Perez D, Ferreira P, Martinez AT, Alcalde M (2015) Focused directed evolution of aryl-alcohol oxidase in Saccharomyces cerevisiae by using chimeric signal peptides. Applied and Environmental Microbiology 81(18):6451–6462. 10.1128/AEM.01966-15 26162870 10.1128/AEM.01966-15 PMC4542223

[CR235] Viña-Gonzalez J, Jimenez-Lalana D, Sancho F, Serrano A, Martinez AT, Guallar V, Alcalde M (2019) Structure-guided evolution of aryl alcohol oxidase from pleurotus eryngii for the selective oxidation of secondary benzyl alcohols. Advanced Synthesis & Catalysis 361(11):2514–2525. 10.1002/adsc.201900134

[CR236] Violante-Mota F, Tellechea E, Moran JF, Sarath G, Arredondo-Peter R (2010) Analysis of peroxidase activity of rice (Oryza sativa) recombinant hemoglobin 1: implications for in vivo function of hexacoordinate non-symbiotic hemoglobins in plants. Phytochemistry 71(1):21–26. 10.1016/j.phytochem.2009.09.016 19833360 10.1016/j.phytochem.2009.09.016

[CR237] Vojinović V, Azevedo AM, Martins VCB, Cabral JMS, Gibson TD, Fonseca LP (2004) Assay of H2O2 by HRP catalysed co-oxidation of phenol-4-sulphonic acid and 4-aminoantipyrine: characterisation and optimisation. Journal of Molecular Catalysis B: Enzymatic 28(2):129–135. 10.1016/j.molcatb.2004.02.003

[CR238] Vojinović V, Carvalho RH, Lemos F, Cabral JMS, Fonseca LP, Ferreira BS (2007) Kinetics of soluble and immobilized horseradish peroxidase-mediated oxidation of phenolic compounds. Biochemical Engineering Journal 35(2):126–135. 10.1016/j.bej.2007.01.006

[CR239] Wang T, Reckhow DA (2016) Spectrophotometric method for determination of ozone residual in water using ABTS: 2.2′-azino-bis (3-ethylbenzothiazoline-6-sulfonate). Ozone: Science & Engineering 38(5):373–381. 10.1080/01919512.2016.1188681

[CR240] Wang Y, Xue P, Cao M, Yu T, Lane ST, Zhao H (2021) Directed evolution: methodologies and applications. Chem Rev 121(20):12384–12444. 10.1021/acs.chemrev.1c00260 34297541 10.1021/acs.chemrev.1c00260

[CR241] Welling GW, Scheffer AJ, Welling-Wester S (1994) Determination of enzyme activity by high-performance liquid chromatography. Journal of Chromatography B: Biomedical Sciences and Applications 659(1):209–225. 10.1016/0378-4347(94)00154-5 10.1016/0378-4347(94)00154-5 7820278

[CR242] Wellner, D., & Lichtenberg, L. A. (1971). [218a] Assay of amino acid oxidase. In *Methods in enzymology* (Vol. 17, pp. 593–596). Academic Press. 10.1016/0076-6879(71)17104-2

[CR243] Werner W, Rey HG, Wielinger H (1970) Über die Eigenschaften eines neuen Chromogens für die Blutzuckerbestimmung nach der GOD/POD-Methode. Fresenius’ Zeitschrift Für Analytische Chemie 252(2):224–228. 10.1007/BF00546391

[CR244] Wesolowski J, Hassan RYA, Hodde S, Bardroff C, Bilitewski U (2008) Sensing of oxygen in microtiter plates: a novel tool for screening drugs against pathogenic yeasts. Anal Bioanal Chem 391(5):1731–1737. 10.1007/s00216-008-1947-6 18297273 10.1007/s00216-008-1947-6

[CR245] Witte DL, Brown LF, Feld RD (1978) Effects of bilirubin on detection of hydrogen peroxide by use of peroxidase. Clin Chem 24(10):1778–1782. 10.1093/clinchem/24.10.1778 699289

[CR246] Wong CM, Wong KH, Chen XD (2008) Glucose oxidase: natural occurrence, function, properties and industrial applications. Appl Microbiol Biotechnol 78(6):927–938. 10.1007/s00253-008-1407-4 18330562 10.1007/s00253-008-1407-4

[CR247] Wong RC, Ngo TT, Lenhoff HM (1981) Formation of blue chromophore from oxidative coupling of aminoantipyrine with chromotropic acid in the presence of peroxide and horseradish peroxidase. International Journal of Biochemistry 13(2):159–163. 10.1016/0020-711X(81)90151-8

[CR248] Yamashita M, Omura H, Okamoto E, Furuya Y, Yabuuchi M, Fukahi K, Murooka Y (2000) Isolation, characterization, and molecular cloning of a thermostable xylitol oxidase from Streptomyces sp. IKD472. Journal of Bioscience and Bioengineering 89(4):350–360. 10.1016/S1389-1723(00)88958-6 16232758 10.1016/s1389-1723(00)88958-6

[CR249] Yang H, Johnson PM, Ko K-C, Kamio M, Germann MW, Derby CD, Tai PC (2005) Cloning, characterization and expression of escapin, a broadly antimicrobial FAD-containing l-amino acid oxidase from ink of the sea hare Aplysia californica. J Exp Biol 208(18):3609–3622. 10.1242/jeb.01795 16155232 10.1242/jeb.01795

[CR250] Yang SY, Schulz H (1987) Kinetics of coupled enzyme reactions. Biochemistry 26(17):5579–5584. 10.1021/bi00391a054 3676269 10.1021/bi00391a054

[CR251] Youdim MBH, Edmondson D, Tipton KF (2006) The therapeutic potential of monoamine oxidase inhibitors. Nat Rev Neurosci 7(4):295–309. 10.1038/nrn1883 16552415 10.1038/nrn1883

[CR252] Zatón AML, Ochoa de Aspuru E (1995) Horseradish peroxidase inhibition by thiouracils. FEBS Letters 374(2):192–194. 10.1016/0014-5793(95)01088-V 7589532 10.1016/0014-5793(95)01088-v

[CR253] Zhang Y, Hess H (2020) Inhibitors in commercially available 2,2′-azino-bis(3-ethylbenzothiazoline-6-sulfonate) affect enzymatic assays. Anal Chem 92(1):1502–1510. 10.1021/acs.analchem.9b04751 31795631 10.1021/acs.analchem.9b04751

[CR254] Zhao B, Ranguelova K, Jiang J, Mason RP (2011) Studies on the photosensitized reduction of resorufin and implications for the detection of oxidative stress with Amplex Red. Free Radical Biology and Medicine 51(1):153–159. 10.1016/j.freeradbiomed.2011.03.016 21419845 10.1016/j.freeradbiomed.2011.03.016 PMC3109108

[CR255] Zhao B, Summers FA, Mason RP (2012) Photooxidation of Amplex Red to resorufin: Implications of exposing the Amplex Red assay to light. Free Radical Biology and Medicine 53(5):1080–1087. 10.1016/j.freeradbiomed.2012.06.034 22765927 10.1016/j.freeradbiomed.2012.06.034 PMC3501008

[CR256] Zhao X, Yu H, Liang Q, Zhou J, Li J, Du G, Chen J (2023) Stepwise optimization of inducible expression system for the functional secretion of horseradish peroxidase in Saccharomyces cerevisiae. J Agric Food Chem 71(9):4059–4068. 10.1021/acs.jafc.2c09117 36821527 10.1021/acs.jafc.2c09117

[CR257] Zheng, L., Zhao, M., Xiao, C., Zhao, Q., & Su, G. (2016). Practical problems when using ABTS assay to assess the radical-scavenging activity of peptides: Importance of controlling reaction pH and time. *Food Chemistry*, *192*, 288–294. 10.1016/j.foodchem.2015.07.015 10.1016/j.foodchem.2015.07.015 26304349

[CR258] Zhou M, Diwu Z, Panchuk-Voloshina N, Haugland RP (1997) A stable nonfluorescent derivative of resorufin for the fluorometric determination of trace hydrogen peroxide: applications in detecting the activity of phagocyte NADPH oxidase and other oxidases. Analytical Biochemistry 253(2):162–168. 10.1006/abio.1997.2391 9367498 10.1006/abio.1997.2391

[CR259] Zhu A, Romero R, Petty HR (2010) Amplex UltraRed enhances the sensitivity of fluorimetric pyruvate detection. Anal Biochem 403(1–2):123–125. 10.1016/j.ab.2010.04.008 20382105 10.1016/j.ab.2010.04.008 PMC2879451

[CR260] Zollner, H. (1999). Enzyme → Inhibitor List: H. In *Handbook of enzyme inhibitors* (Vol. 3, pp. 2191–2273). WILEY-VCH Verlag GmbH. 10.1002/9783527618330.ch34

